# Differential Response to NaCl Osmotic Stress in Sequentially Harvested Hydroponic Red and Green Basil and the Role of Calcium

**DOI:** 10.3389/fpls.2022.799213

**Published:** 2022-03-09

**Authors:** Michele Ciriello, Luigi Formisano, Georgios A. Soteriou, Angelos Kyratzis, Stefania De Pascale, Marios C. Kyriacou, Youssef Rouphael

**Affiliations:** ^1^Department of Agricultural Sciences, University of Naples Federico II, Naples, Italy; ^2^Department of Vegetable Crops, Agricultural Research Institute, Nicosia, Cyprus

**Keywords:** *Ocimum basilicum* L., nutritional stress, successive harvests, isomolar concentrations, volatile compounds, calcium chloride

## Abstract

Basil (*Ocimum basilicum* L.) is a heterogeneous reservoir of bioactive compounds that provide recognized benefits to human health, rendering it a model aromatic herb. Notwithstanding the application of nutritional stress, such as sodium chloride (NaCl) salinity, which mainly affects the primary metabolism, it also triggers adaptive mechanisms that involve the production of bioactive secondary metabolites. Genotype selection and the exogenous application of calcium chloride (CaCl_2_) help minimize salinity’s suppressive effects on growth. In the present study, we hypothesize that the ratio of different salt types may induce differential responses in the function of preharvest factors in hydroponic basil culture. In this perspective, the stock nutrient solution (Control) was supplemented with 12.5 mm NaCl + 8.33 mm CaCl_2_ (Moderate Mix), 25 mm NaCl (Moderate NaCl), 25 mm NaCl + 16.66 of CaCl_2_ (High Mix), or 50 mM of NaCl (High NaCl) with the objective of evaluating the different impact of salinity on yield, sensory quality (color and aroma profile), and the accumulation of minerals and bioactive compounds in two successive harvests of green and red basil cultivars. Although more productive (+39.0% fresh weight) than the red one, the green cultivar exhibited higher susceptibility to salinity, especially under the High Mix and High NaCl treatments. The addition of CaCl_2_ to the High Mix solution reduced the sodium by 70.4% and increased the total polyphenols by 21.5% compared to the equivalent isomolar solution (High NaCl). The crop performance in terms of fresh and dry yield improved for both cultivars at the second cut. Regardless of cultivar and salt treatment, successive harvests also increased the concentration of phenols and vitamin C (29.7 and 61.5%, respectively) while reducing (−6.9%) eucalyptol, the most abundant aromatic compound in both cultivars. Salinity, as well as the mechanical stress induced by cutting, improved the functional quality of basil. However, the productive responses to the conditions imposed in our work once again highlighted the importance of genetic background. Specifically, CaCl_2_ in the Moderate Mix solution preserved fresh leaf weight in the most stress-sensitive green cultivar.

## Introduction

Basil (*Ocimum basilicum* L.) is a native herbaceous plant of southern Asia, also known as the “king of herbs” for its fragrant, delightful, and colorful leaves (Ciriello et al., 2020; Formisano et al., 2021b). As a culinary herb, it is preferably used fresh to improve the aroma and appearance of salads, pizza, meat, and fish dishes (Corrado et al., 2020a,b). According to vernacular knowledge, basil has wound-healing properties and its leaves and flowering tops are used for preparing infusions claimed to have sedative, antispasmodic, stomachic, diuretic, and antimicrobial effects (Ahmed et al., 2019; Amor et al., 2021). Furthermore, it is also used to treat indigestion and as a vermifuge due to its bioactive content and high nutraceutical value (Ch et al., 2015). These secondary metabolites of aromatic herbs are a precious reservoir of bioactive molecules that the pharmaceutical industry could use to develop new drugs and improve their natural chemical diversity (David et al., 2015). In this respect, recent scientific research has highlighted how genotype and agronomic practices, such as successive harvests, can be a valuable tool toward increasing the levels of desired secondary metabolites (Nicoletto et al., 2013; Ciriello et al., 2021a,b).

In the previous three decades, research efforts were focused mainly on boosting the desirable phytochemicals (e.g., ascorbate, carotenoids, glucosinolates, polyamines, and polyphenols) in fruits and vegetables through plant breeding and biotechnology, but have yet achieved limited commercial success because of safety issues (Poiroux-Gonord et al., 2010). For instance, the disproportional increase in some secondary metabolites generated through breeding and biotechnology (e.g., a 36-fold increase in kaempferol-rutinoside, a 20-fold increase in carotenoids, and glucosinolates) has been found genotoxic (Rouphael and Kyriacou, 2018). Moreover, the improvement of plant phytochemicals through breeding constitutes a costlier approach than crop management practices. Therefore, the manipulation of phytochemicals through controlled agro-environmental conditions (air and root zone temperature, light quality and intensity, carbon dioxide enrichment, and vapor pressure deficit), as well as innovative crop management practices (nutritional eustress, nutrient solution management, and biofortification) constitutes an approach balancing safety, cost, and effectiveness (Poiroux-Gonord et al., 2010). Unsurprisingly, this approach has attracted the interest of food technologists and extension specialists as it additionally quells consumers’ concerns over genetic modification of food products (Kyriacou and Rouphael, 2018). In this perspective, nutrient solution management posits as a significant toolbox for improving the functional quality of vegetal food (Rouphael and Kyriacou, 2018). As demonstrated in the current work, soilless culture may facilitate the effective and precise control of the plant-nutrient interface to achieve target concentrations in key secondary metabolites (Fallovo et al., 2009a,b; Colla et al., 2013a; Borgognone et al., 2016). Moreover, accurate application of chemical eustress without soil interaction, such as the controlled deprivation of macronutrients, exposure to mild or moderate salinity [Sodium Chloride (NaCl)], and sustained exposure of the roots to variable cationic and anionic proportions or single ions, may boost the bioactive phytochemical content of vegetables within the optimal range (Rouphael and Kyriacou, 2018).

A prerequisite to achieving these targets is to identify the optimal balance between exposure time and phenological stage of stress application, as there is strong crop intraspecific and interspecific genetic variability toward stress tolerance (Rouphael et al., 2018). Moderate salt concentrations in the soil solution effectively trigger metabolic pathways involved in the production of secondary metabolites in the context of a concerted adaptive mechanism against oxidative damage induced by salinity (Tarchoune et al., 2013). However, salinity levels exceeding the crop’s tolerance threshold can induce morpho-physiological and metabolic changes capable of retarding and/or inhibiting plant growth (Ntatsi et al., 2017). Salinity damage is primarily co-induced by excess NaCl, which jointly triggers osmotic (low water potential) and ion stress (toxicity and ion-specific imbalance) (Munns and Tester, 2008; Colla et al., 2013b). The combination of both stressors leads to chlorophyll and carotenoids pigment degradation, stomatal closure, and photosystem II (PSII) blockage, with a consequent reduction in carbon organization and impaired translocation and uptake of critical nutrients (das Neves et al., 2008; Liu et al., 2012).

Calcium, due to its role as a structural component of membranes and cell walls, is crucial for plant survival under suboptimal environmental conditions (Colla et al., 2013b; Cheruth et al., 2016). Its regulatory role in nutrient uptake processes, implicated in ensuring the proper integrity and permeability of cell membranes, mitigates the ionic effect of NaCl (Borgognone et al., 2014a). Several authors have suggested that the exogenous application of calcium may alleviate the harmful effects induced by excess NaCl in model crops such as wheat (*Triticum aestivum L.*), tomatoes (*Solanum lycopersicum* L.), peas (*Pisum sativum* L. cv. Argona), and sunflowers (*Helianthus annuus* L.) (Bonilla et al., 2004; Zhou and Xiao, 2010; Borgognone et al., 2014a). Specifically, calcium drives ion-selective transport and maintains the key K/Na and Ca/Na ratios at optimal levels by reducing the cytosolic Na uptake (Nedjimi and Daoud, 2009).

Most studies on the salt stress tolerance of vegetable crop species have focused on the role of NaCl as the predominant salt (Bernstein et al., 2010; Zahedi et al., 2011; Barbieri et al., 2012). Only a few of these studies have additionally evaluated the calcium-induced ameliorative effects on the growth and physiology of crops, such as cucumber (*Cucumis sativus* L. cv. Palmera) (Trajkova et al., 2006), basil (*Ocimum basilicum* L. cv. Thai Siam Queen) (Scagel et al., 2017), marjoram (*Satureja montana)* (Hegazy et al., 2019), chicory (*Cichorium spinosum*) (Ntatsi et al., 2017), lettuce (*Lactuca sativa* L. cv. Lollo Rossa) (Borghesi et al., 2013; Cheruth et al., 2016), sorghum (*Sorghum bicolor* L. Monench, cv. ICI-5521) (Dashti et al., 2009), and wheat (Nemat Alla et al., 2014). However, these authors mainly used variable NaCl molar concentrations without distinguishing the osmotic from the ionic effect and without assessing the natural sensitivity of the cultures to sodium (Na) and chlorine (Cl). To date, few studies have investigated the ameliorative effects of CaCl_2_ on NaCl-induced salt stress using isomolar concentrations of the two salts (Colla et al., 2013b; Borgognone et al., 2014a,b).

In the present study, we hypothesize that not only different kinds of salt but also their ratio may induce differentiated responses in basil crops. Understanding these responses could help growers pursue appropriate agricultural practices to improve plant tolerance to salinity, enhance the plant secondary metabolome and enable efficient production under prevailing suboptimal conditions. Based on this hypothesis, the objective of the present work was to evaluate the effect of two salts (NaCl and CaCl_2_), added at isomolar concentrations to the stock nutrient solution (Control) in a hydroponic greenhouse system, with respect to the growth, quality, and volatile aromatic components of green (cv. Mammolo) and red (cv. Red Rubin) basil (*O. basilicum* L.), harvested twice during the crop cycle (first and second cut).

## Materials and Methods

### Experimental Site and Design and Management of the Nutrient Solution

The experimental trial was carried out at the “Torre Lama” experimental farm of the University of Naples Federico II – Department of Agriculture (DIA) located in Bellizzi (SA, Italy; lat. 43° 31′ N, long. 14° 58′ E; alt. 60 m above sea level). The trial was conducted in a glass greenhouse 10 m wide, 30 m long, and 3 and 4.5 m high at the eaves and ridge, respectively, from April 29, 2015, to July 2, 2015. Twenty days after sowing (April 29, 2015; DAT 1), two basil (*O. basilicum* L.) cultivars with different pigmentation “Mammolo” (hereafter “Green”; L’ortolano, Cesena, FC, Italy) and “Red Rubin” (hereafter “Red”; Ortomio, Forlì, FC, Italy) were transplanted into square anti-spiral pots (0.1 m × 0.1 m × 0.15 m) filled with 1.3 L volume of a mixture (*v/v*) consisting of 1/3 perlite (Vigorplant, Fombio, LO, Italy) and 2/3 peat (Vigorplant, Fombio, LO, Italy). The seedlings were arranged in rows with a spacing of 0.30 m × 0.15 m yielding a density of 22 plants m^–2^.

The plants were arranged according to a factorial design with three replicates in which a non-saline control (Control) vs. four saline nutrient solutions (Moderate Mix, Moderate NaCl, High Mix, High NaCl), two basil cultivars (Green and Red), and two successive harvests (Cut1 and Cut2) were considered as factors. Each experimental unit (i.e., replicate) included 15 plants. The standard nutrient solution (NS; Control) was a Hoagland modified as follows: 13.0 mM NO_3_-N, 1.0 mM NH_4_-N, 1.75 mM S, 1.5 mM P, 5.0 mM K, 4.5 mM Ca, 2 mM Mg, 20 μM Fe, 9 μM Mn, 0.3 μM Cu, 1.6 μM Zn, 20 μM B, and 0.3 μM Mo. Saline NS were prepared by adding isomolar concentrations of NaCl and CaCl_2_ to the standard NS (Table 1). The NS was prepared using osmotized water and distributed through a drip irrigation system (1 dripper/plant with a flow rate of 2 L/h). To avoid large fluctuations in EC, pH, and the accumulation of salts in the substrates, for each pot, additional irrigations with water were provided to drain the excess salts, keeping the variation of EC within a value never higher or lower than 5% of the initial value.

**TABLE 1:** Total ion concentration and sodium chloride (NaCl) and calcium chloride (CaCl_2_) concentration in saline nutrient solutions.

Treatment	NaCl	CaCl_2_	Total ion concentration
	mM	mM	mM
Control	0	0	0
Moderate Mix	12.5	8.33	50
Moderate NaCl	25	0	50
High Mix	25	16.66	100
High NaCl	50	0	100

### Growth, Yield, and CIELab Colorimetric Index Measurements

At 42 and 65 days after transplanting (DAT) (June 9-Cut1 and July 2-Cut2), and just before flowering, the plants were harvested. At each cut, 10 plants per experimental unit were sampled, immediately weighed, and the leaves were separated. The fresh weight (FW), number, and total leaf area were determined using the LI-COR 3100C area meter (LI-COR Biosciences, Lincoln, NE, United States). The plant tissues were dried in a ventilated oven at 65°C for 72 h for total and leaf dry weight (DW) determination; the latter was used to determine leaf dry matter (DM) percentage (%) as follows: DM = 100 × leaf DW/leaf FW. Samples were finely ground and sieved with a cutting-grinding head mill (IKA^®^, Staufen im Breisgau, BW, Germany). An aliquot of fresh ground leaves was immediately placed in liquid nitrogen and stored at −80°C for future qualitative analysis.

On the same dates (42 and 65 DAT), colorimetric measurements of the CIELab color space were made on the adaxial surface of 30 healthy and fully expanded leaves for treatment using a portable Minolta CR-300 Chroma Meter (Minolta Camera Co., Ltd., Tokyo, Kantō, Japan), calibrated according to the manufacturer’s instructions. As defined by the Commission Internationale de l’Éclairage (CIE), color was expressed according to the three components L* (lightness), a* (greenness), and b* (yellowness) by which the Chroma (C*) and Hue angle (h°) values were determined.

### Total Nitrogen, Nitrate, and Macronutrient Determination

Finely ground dry leaf samples were analyzed by the Kjeldahl method for total nitrogen determination and by ion chromatography for macronutrients [phosphorus (P), sulfur (S), potassium (K), calcium, magnesium (Mg), sodium, and chlorine] and nitrate, following the protocols described by Bremner (1965) and Formisano et al. (2021a), respectively.

Briefly, for the total nitrogen determination, 0.25 g of sample was mixed with 7 mL of 96% H_2_SO_4_ (Carlo Erba Reagents Srl., Milan, MI, Italy), a catalyst (Velp^®^ Scientifica, Usmate Velate, MB, Italy), and 10 mL of 30% H_2_O_2_ (Carlo Erba Reagents Srl., Milan, MI, Italy). The obtained mixture was heated at 420°C with a DK 20 Heating Digester (Velp^®^ Scientifica, Usmate Velate, MB, Italy) until mineralization (30 min). Mineralized samples were distilled with a UDK 140 distiller (Velp^®^ Scientifica, Usmate Velate, MB, Italy) and subsequently titrated with H_2_SO_4_ 0.1 N with a methyl red and bromocresol green indicator (Carlo Erba Reagents Srl., Milan, MI, Italy) until the solution reached reddish color. The total nitrogen concentration values were expressed as g kg^–1^ of dry weight (DW). Each treatment was analyzed in triplicate. The results obtained on the quantification of minerals, antioxidant activity, total phenols, and ascorbic acid (AsA) were expressed in terms of concentration according to the definition proposed by Bauer et al. (1997).

For the determination of macronutrient and nitrate concentrations, 0.25 g of sample was extracted in ultrapure water (Arium^®^ Advance EDI pure water system, Sartorius, Göttingen, NI, Germany) and analyzed using an ICS-3000 ion chromatographic system (Thermo Scientific™ Dionex™, Sunnyvale, CA, United States), coupled with an electrical conductivity detector. The determination of anions (nitrate, sulfate, P, and Cl) and cations (K, calcium, Mg, and sodium) was performed by isocratic and gradient mode separation, respectively. The concentration of anions and cations in leaves were expressed as g kg^–1^ of DW, except for nitrate expressed as μg g^–1^ of FW. Each treatment was analyzed in triplicate.

### Antioxidant Activity Determination, Total Phenols, and Total Ascorbic Acid

The determination of the antioxidant 2,2′-azino-bis(3-ethylbenzothiazoline-6-sulfonic acid) (ABTS) fraction was determined according to the method described by Pellegrini et al. (1998). This method is based on chromogen 2,2′-azino-bis(3-ethylbenzothiazoline-6-sulfonic acid), which registers in its radical form an absorption maximum at 734 nm. The ABTS antioxidant activity was expressed as g Trolox eq kg^–1^ of DW.

The quantification of the total phenolic compounds was determined according to Folin-Ciocalteu’s method described by Singleton et al. (1999). Briefly, 0.25 g of freeze-dried and ground leaves were added to 10 ml of 60% methanol, placed on a tilting plate for 15 min, and centrifuged. Subsequently, 125 μl of the supernatant was taken, and 125 μl of a mixture of phosphotungstic acid (H_3_PW_12_O_40_) and phosphomolybdic acid (H_3_Mo_12_PO_40_) (Folin-Ciocalteu reagent) was added to 0.5 mL of distilled water. In the presence of phenolic compounds, the Folin-Ciocalteu reagent is reduced to a mixture of tungsten (W_8_O_23_) and molybdenum (Mo_8_O_23_) oxides that has an absorption peak at 760 nm. The total phenolic concentration was expressed as mg gallic acid equivalents 100 g^–1^ DW.

The total AsA concentration was determined by an assay based on the reduction of Fe^3+^ to Fe^2+^ by reduced AsA and the spectrophotometric detection of Fe^2+^ with 2,2′-dipyridyl (Kampfenkel et al., 1995). An aliquot of 0.4 g of frozen sample was weighed and then crushed in a mortar with 0.8 mL of 6% trichloroacetic acid (TCA). After incubation for 15 min at −20°C, 1.2 mL of 6% TCA was added to the extract, after which it was centrifuged at 4,000 rpm for 10 min. The obtained supernatant was taken to determine total AsA (AsA + dehydroascorbate). The absorbance was then read at a wavelength of 525 nm, which corresponds to the maximum absorption peak of the Fe/2,2-dipyridyl.

UV-vis spectrophotometry (Hach DR 4000; Hach Co., Loveland, CO, United States) was used to read the absorbances of antioxidant activities, total phenols, and AsA. Each treatment was analyzed in triplicate.

### Volatile Compounds Determination

In order to quantify volatile compounds (VOCs) *via* the solid-phase microextraction (SPME) method, 0.5 g of basil leaves stored at −80°C were manually crushed and placed in a 20 ml glass vial that was shaken and heated for 10 min to promote the migration of VOCs into the headspace. The VOCs were absorbed by a divinylbenzene/carboxen/polydimethylsiloxane fiber (1 cm long, 50/30 μm thick; Supelco^®^, Bellefonte, PA, United States). VOCs were determined by GC 6890N gas chromatograph (Agilent, Santa Clara, CA, United States) coupled with MS 5973N mass spectrometer (Agilent, Santa Clara, CA, United States) equipped with a split-splitless injector and a capillary column (30 m × 0.250 mm) coated with a 5% phenyl/95% dimethylpolysiloxane film (0.25 μm; Supelco^®^, Bellefonte, PA, United States).

Following a desorption phase of 10 min at 230°C, the oven temperature was kept at 50°C for 2 min and then ramped to 150°C at 10°C/min and from 150 to 280 at 15°C/min. The ion and injection source temperatures were 230 and 250°C, respectively. Helium was used as the carrier gas at a flow rate of 1 ml min^–1^. The mass spectrometer was set at 70 eV. The identification of VOCs was facilitated through the use of the Atomic Spectra Database version 1.6 of the National Institute of Standards and Technology (NIST; U.S. Department of Commerce, Gaithersburg, MD, United States). Samples were analyzed in triplicates and the results were expressed as a percentage relative abundance (%).

### Statistics

The experiment was set up in a randomized design (Completely Randomized Design) with three replicate plots per treatment. Thirty (2 cultivars × 5 salinity levels × 3 replications) fully randomized experimental plots were used for each cut (Cut 1 and Cut 2). Each experimental plot accommodated 15 plants. During each cut, all the 15 plants of each experimental plot were harvested. Following sampling, aggregates of 15 plants were made for each treatment replication. Therefore 450 plants (30 experimental plots × 15 plants) were used during each cut and 900 plants for the whole study (Cut1 and Cut2). ANOVA was performed to assess the significance of the main effects [Cultivar (V), Salinity (S), Cut (C)] and their pair-wise interactions. During the statistical analysis, we ensured that all the assumptions of ANOVA were met. That is, the treatment replications samples were independent of each other, sample populations were normally distributed (normality), and the sample populations had the same variance (homoscedasticity). Three-way interactions were either non-significant or had a very low contribution to the total variance of the examined variables. Means for V and C were compared by the Student’s *t*-test, whereas the means for S and all two-way interactions were compared using the Tukey–Kramer HSD test at the *p* < 0.05 level. Data represent mean ± SE of three replicates (*n* = 3). All statistical analyses were performed using IBM SPSS 20 (Armonk, NY, United States) package for Microsoft Windows 10.

## Results

### Morphometric and Yield Parameters

With respect to the main experimental factors, significant differences were observed between cultivars for leaf number, leaf area, and leaf FW (Table 2). Superior leaf number but smaller leaf area and leaf FW were observed in Red, reflecting the smaller leaves of this cultivar compared to Green, whereas leaf DM percentage and DW were not influenced by cultivar type. Salinity treatments had no effect on the leaf number and leaf DM percentage; however, the High Mix treatment and the High NaCl treatment suppressed the leaf area, leaf FW, and DW, which signals a deleterious effect on plant growth under both high-salt treatments. Cut order affected significantly all the morphometric variables examined, with leaf number, area and FW, and DW being higher at Cut2, at the expense, however, of leaf DM percentage. Significant salinity × cultivar interaction was observed for leaf area, leaf FW, and DW as the mean values of Red for these variables under the High Mix and High NaCl treatments did not differ significantly to the Control (non-salt) treatment. The rest of the interactions observed derived from changes in the scale of the observed effects but not in the ranking of treatments.

**TABLE 2:** Analysis of variance (ANOVA) and mean comparisons for leaf number, leaf area, leaf fresh weight, leaf dry matter percentage, and dry weight of the Green and Red cultivars grown hydroponically under iso-osmotic salinity treatments and harvested in two sequential cuts.

Source of variance	Leaf number	Leaf area	Leaf FW	Leaf DM	Dry weight
	n° plant^–1^	(cm^2^ plant^–1^)	(g plant^–1^)	(%)	(g plant^–1^)
Cultivar (V)	***	*	***	ns	ns
Salinity (S)	ns	***	***	ns	***
Cut (C)	***	***	***	*	***
S × V	ns	***	***	ns	*
S × C	ns	ns	ns	ns	*
V × C	***	**	*	*	**

	**Means ± standard error**				
	
**Cultivar**					
Green	122 ± 8b	1639 ± 89a	60.72 ± 3.39a	9.00 ± 0.20	7.96 ± 0.42
Red	197 ± 14a	1482 ± 79b	43.40 ± 2.48b	9.18 ± 0.14	7.27 ± 0.48
**Salinity**					
Control	159 ± 17	1862 ± 127a	64.05 ± 6.56a	8.58 ± 0.16	8.73 ± 0.83a
Moderate Mix	185 ± 22	1893 ± 73a	63.44 ± 3.72a	8.88 ± 0.21	9.15 ± 0.54a
Moderate NaCl	159 ± 23	1657 ± 130a	54.16 ± 4.08ab	9.22 ± 0.28	8.07 ± 0.75ab
High Mix	148 ± 21	1291 ± 97b	43.38 ± 3.64bc	8.94 ± 0.29	6.36 ± 0.47bc
High NaCl	138 ± 18	1042 ± 109b	34.15 ± 2.96c	9.88 ± 0.32	5.51 ± 0.47c
**Cut**					
Cut1	101 ± 5b	1287 ± 69b	42.96 ± 2.69b	9.21 ± 0.14a	5.89 ± 0.29b
Cut2	219 ± 11a	1853 ± 76a	62.09 ± 3.16a	8.96 ± 0.20b	9.44 ± 0.41a
**S × V**					
Control × Green	136 ± 20	2229 ± 126a	85.90 ± 5.70a	8.25 ± 0.18	10.66 ± 1.12a
Moderate Mix × Green	130 ± 19	1951 ± 93ab	73.02 ± 3.98ab	8.38 ± 0.20	9.09 ± 0.69abc
Moderate NaCl × Green	118 ± 18	1783 ± 142abcd	60.38 ± 3.63bc	8.90 ± 0.38	7.83 ± 0.69abc
High Mix × Green	113 ± 15	1286 ± 91def	48.40 ± 4.53cde	9.01 ± 0.47	6.57 ± 0.42bc
High NaCl × Green	111 ± 16	947 ± 130f	35.89 ± 3.93e	10.45 ± 0.48	5.64 ± 0.49c
Control × Red	182 ± 27	1494 ± 119bcde	42.20 ± 3.97de	8.91 ± 0.22	6.81 ± 0.78bc
Moderate Mix × Red	239 ± 29	1835 ± 115abc	53.86 ± 4.14cd	9.38 ± 0.28	9.21 ± 0.87ab
Moderate NaCl × Red	200 ± 38	1532 ± 219bcde	47.94 ± 6.87cde	9.54 ± 0.41	8.31 ± 1.39abc
High Mix × Red	191 ± 35	1336 ± 176cdef	38.90 ± 5.29de	8.81 ± 0.30	6.29 ± 0.91bc
High NaCl × Red	170 ± 31	1151 ± 181ef	32.15 ± 4.70e	9.23 ± 0.25	5.37 ± 0.88c
**S × C**					
Control × Cut1	101 ± 9	1616 ± 174	54.12 ± 8.50	8.62 ± 0.20	6.81 ± 0.79bc
Moderate Mix × Cut1	126 ± 18	1683 ± 82	54.88 ± 4.35	9.14 ± 0.26	7.65 ± 0.53b
Moderate NaCl × Cut1	91 ± 11	1251 ± 122	41.75 ± 4.59	9.36 ± 0.33	5.77 ± 0.54bcd
High Mix × Cut1	98 ± 9	1056 ± 57	35.44 ± 3.08	9.15 ± 0.35	5.05 ± 0.23cd
High NaCl × Cut1	91 ± 8	828 ± 34	28.62 ± 1.84	9.76 ± 0.28	4.20 ± 0.19d
Control × Cut2	218 ± 15	2107 ± 145	73.98 ± 9.18	8.53 ± 0.27	10.66 ± 1.11a
Moderate Mix × Cut2	244 ± 27	2103 ± 58	72.00 ± 4.40	8.61 ± 0.32	10.64 ± 0.56a
Moderate NaCl × Cut2	227 ± 28	2064 ± 102	66.57 ± 2.51	9.09 ± 0.48	10.38 ± 0.79a
High Mix × Cut2	208 ± 30	1599 ± 107	53.70 ± 4.58	8.64 ± 0.45	8.03 ± 0.45ab
High NaCl × Cut2	192 ± 25	1288 ± 197	40.45 ± 5.19	10.02 ± 0.62	7.01 ± 0.59bc
**V × C**					
Green × Cut1	77 ± 3d	1442 ± 110b	53.28 ± 3.80b	8.97 ± 0.20ab	6.49 ± 0.42b
Green × Cut2	166 ± 5b	1837 ± 129a	68.15 ± 5.18a	9.03 ± 0.35ab	9.42 ± 0.57a
Red × Cut1	126 ± 7c	1132 ± 70c	32.64 ± 1.98c	9.45 ± 0.17a	5.30 ± 0.37b
Red × Cut2	277 ± 13a	1870 ± 76a	55.36 ± 2.73b	8.89 ± 0.20b	9.47 ± 0.59a

Salinity treatments: Control = non-salt; Moderate Mix = (12.5 mM NaCl + 8.3 mM CaCl_2_); Moderate NaCl = 25 mM NaCl; High Mix = (25 mM NaCl + 16.6 mM CaCl_2_); High NaCl = 50 mM NaCl. * Significant effect at the 0.05 level, **0.01 level, *** 0.001 level, ns = non-significant effect. Data represent means ± standard error of 3 replicates (n = 3) consisting of 10 plants each. Treatment means within each column followed by different letters denote significant differences (p < 0.05) according to Tukey–Kramer HSD test. Three-way interactions were either non-significant or had a very low contribution to the total variance of the examined variables.

### Leaf Colorimetric Components

Significant differences were observed between cultivars for the leaf colorimetric attributes examined, which largely reflects their apparent genotypic differences in color (Table 3). Lightness (L*) was overall higher in Green than Red and in Cut2 than Cut1, while slightly higher L* was also observed with salinity treatments compared to Control. Cultivar ranking did not change for L* across salinity treatments as the salinity × cultivar interaction observed was due to change in scale rather than treatment rank. Similarly, cut ranking was invariable across salinity treatments despite salinity × cut interaction. However, the significant cultivar × cut interaction observed highlighted that leaf L* of Green remained unaltered between Cut1 and Cut2, as opposed to the sharp increase observed in Red. Significant variation in response to salinity treatments was only observed for Red as the red color intensity (+a*) decreased significantly with all salinity treatments, except for Moderate Mix when compared to the Control. Moreover, under both moderate and high salinity conditions, the NaCl treatment tended to suppress red color intensity more than the combined NaCl + CaCl_2_ treatment. Significant cultivar × cut interaction was also observed as the green color intensity (−a*) of green basil was not dependent on the cut, whereas the red color intensity (+a*) of Red decreased at Cut2. With respect to the overall leaf color saturation or chroma (C*), Green demonstrated higher C* than Red irrespective of salinity and cut. The cut order was not significant for leaf chroma in Green; however, Red demonstrated an increase in leaf chroma at Cut2.

**TABLE 3:** Analysis of variance (ANOVA) and mean comparisons for leaf colorimetric components L*, a*, b*, Chroma (C*), and hue Angle (h°) of the Green and Red cultivars grown hydroponically under iso-osmotic salinity treatments and harvested in two sequential cuts.

Source of variance	L*	a*	b*	C*	h°
	(0–100)	(−60/+60)	(−60/+60)	√(a^2^ + b^2^)	(0–360°)
Cultivar (V)	***	***	***	***	***
Salinity (S)	***	*	ns	ns	*
Cut (C)	***	***	*	ns	***
S × V	***	***	***	*	*
S × C	***	ns	ns	ns	*
V × C	***	***	***	***	***

	**Means ± standard error**				
	
**Cultivar**					
Green	46.37 ± 0.19a	−6.17 ± 0.11b	13.28 ± 0.36a	14.67 ± 0.37a	115.43 ± 0.28b
Red	40.68 ± 0.58b	2.12 ± 0.33a	3.64 ± 0.48b	5.71 ± 0.35b	186.54 ± 5.70a
**Salinity**					
Control	42.38 ± 1.25b	−1.26 ± 1.40a	7.35 ± 1.91	10.60 ± 1.47	148.75 ± 9.29a
Moderate Mix	43.43 ± 1.11ab	−1.75 ± 1.18ab	8.20 ± 1.45	9.90 ± 1.38	159.87 ± 12.34a
Moderate NaCl	44.18 ± 0.90a	−2.34 ± 1.10ab	9.03 ± 1.29	10.23 ± 1.35	156.41 ± 12.77a
High Nix	43.95 ± 0.96a	−2.27 ± 1.09ab	9.03 ± 1.20	10.59 ± 1.10	143.11 ± 10.28a
High NaCl	43.68 ± 0.68a	−2.47 ± 0.83b	8.52 ± 1.00	9.45 ± 1.07	146.91 ± 10.59a
**Cut**					
Cut1	41.84 ± 0.71b	−1.59 ± 0.78a	7.97 ± 0.95b	9.77 ± 0.88a	159.47 ± 7.86a
Cut2	45.21 ± 0.37a	−2.46 ± 0.63b	8.94 ± 0.80a	10.61 ± 0.71a	142.49 ± 5.65b
**S × V**					
Control × Green	46.84 ± 0.49a	−6.61 ± 0.30d	14.48 ± 1.02a	15.93 ± 1.05a	114.95 ± 0.72c
Moderate Mix × Green	47.17 ± 0.25a	−6.23 ± 0.29d	13.48 ± 0.96a	14.87 ± 0.98a	115.20 ± 0.70c
Moderate NaCl × Green	46.11 ± 0.25a	−6.45 ± 0.17d	13.72 ± 0.63a	15.18 ± 0.64a	115.41 ± 0.55c
High Mix × Green	46.30 ± 0.35a	−6.01 ± 0.17d	12.82 ± 0.54a	14.18 ± 0.55a	115.38 ± 0.45c
High NaCl × Green	45.42 ± 0.49a	−5.54 ± 0.12d	11.89 ± 0.67a	13.18 ± 0.64a	116.19 ± 0.73c
Control × Red	37.92 ± 0.86c	4.09 ± 0.34a	0.23 ± 0.22c	5.27 ± 0.31b	182.56 ± 6.52ab
Moderate Mix × Red	39.70 ± 1.12c	2.74 ± 0.42ab	2.91 ± 0.33bc	4.93 ± 0.30b	204.54 ± 9.08ab
Moderate NaCl × Red	42.26 ± 1.52b	1.77 ± 0.57bc	4.34 ± 0.71b	5.29 ± 0.64b	197.41 ± 14.79a
High Mix × Red	41.58 ± 1.50b	1.40 ± 1.11bc	5.55 ± 1.57b	7.32 ± 1.38b	170.58 ± 15.40b
High NaCl × Red	41.94 ± 0.94b	0.60 ± 0.46c	5.14 ± 0.74b	5.72 ± 0.69b	177.62 ± 14.50ab
**S × C**					
Control × Cut1	41.33 ± 2.13e	−1.09 ± 2.11	7.50 ± 2.80	10.57 ± 2.19a	152.31 ± 14.71abc
Moderate Mix × Cut1	42.15 ± 2.04de	−1.38 ± 1.85	8.03 ± 2.07	9.77 ± 1.94a	166.97 ± 19.83a
Moderate NaCl × Cut1	42.04 ± 1.42de	−1.79 ± 1.85	8.71 ± 2.27	10.08 ± 2.32a	157.18 ± 20.44a
High Mix × Cut1	41.69 ± 1.50e	−1.51 ± 1.85	7.92 ± 2.26	9.67 ± 2.12a	161.57 ± 17.94ab
High NaCl-Cut1	41.97 ± 0.97de	−2.19 ± 1.39	7.69 ± 1.61	8.75 ± 1.72a	159.35 ± 18.87a
Control × Cut2	43.42 ± 1.34cd	−1.43 ± 1.98	7.21 ± 2.78	10.63 ± 2.13a	145.2 ± 12.23abc
Moderate Mix × Cut2	44.71 ± 0.82bc	−2.12 ± 1.60	8.37 ± 2.17	10.04 ± 2.09a	152.77 ± 15.66abc
Moderate NaCl × Cut2	46.33 ± 0.37a	−2.89 ± 1.29	9.35 ± 1.41	10.39 ± 1.56a	155.64 ± 16.78ab
High Mix × Cut2	46.19 ± 0.51ab	−3.11 ± 1.26	10.45 ± 0.98	11.83 ± 0.86a	124.39 ± 5.88c
High NaCl-Cut2	45.40 ± 0.45ab	−2.74 ± 0.99	9.34 ± 1.21	10.15 ± 1.34a	134.47 ± 8.95bc
**V × C**					
Green × Cut1	46.03 ± 0.30a	−6.35 ± 0.09c	13.73 ± 0.31a	15.14 ± 0.32a	114.90 ± 0.22c
Green × Cut2	46.70 ± 0.21a	−5.99 ± 0.20c	12.82 ± 0.64a	14.02 ± 0.66a	115.95 ± 0.49c
Red × Cut1	37.64 ± 0.34c	3.17 ± 0.27a	2.20 ± 0.33c	4.39 ± 0.20c	204.05 ± 6.68a
Red × Cut2	43.72 ± 0.54b	1.07 ± 0.51b	5.07 ± 0.78b	7.02 ± 0.54b	169.03 ± 7.51b

Salinity treatments: Control = non-salt; Moderate Mix = (12.5 mM NaCl + 8.3 mM CaCl_2_); Moderate NaCl = 25 mM NaCl; High Mix = (25 mM NaCl + 16.6 mM CaCl_2_); High NaCl = 50 mM NaCl. * Significant effect at the 0.05 level, *** 0.001 level, ns = non-significant effect. Data represent means ± standard error of 3 replicates (n = 3) consisting of 10 plants each. Treatment means within each column followed by different letters denote significant differences (p < 0.05) according to the Tukey–Kramer HSD test. Three-way interactions were either non-significant or had a very low contribution to the total variance of the examined variables.

### Leaf Mineral Concentration

The choice of cultivar had a significant effect on the leaf concentration for all minerals analyzed. The cultivar was the only factor with a significant effect on leaf N concentration, which was found higher in Green than in Red (Table 4). P concentration was also higher in Green than Red, however, in the former cultivar, it decreased at Cut2 whereas in the latter it did not, as manifested in significant cultivar × cut interaction. Salinity also had a significant effect on P concentration, which decreased in response to the High Mix and High NaCl treatments. For P, the interaction between the factors under consideration (salinity × cultivar × cut) was significant (*p* ≤ 0.01). In general, the lowest phosphorus concentration was obtained from the High NaCl × Green × Cut2 interaction (data not shown). For the same cultivar in the High Mix and Low Mix treatments, from the first to the second cut, phosphorus decreased by 40.7 and 37.1%, respectively (data not shown). S concentration incurred a significant effect with respect to the cultivar, as it was higher in Red than Green, however, it was not significantly affected by salinity or cut. K concentration was affected by cultivar and cut but not by salinity; it was found higher in Green and increased at Cut2. Calcium, Mg, sodium, and chlorine concentrations were the ones most affected by salinity treatments. Calcium concentration was lowest in the Control and highest in the High-Mix treatment, with the mix treatments resulting in higher Ca concentration than the corresponding isomolar NaCl treatments. The Ca concentration was higher in Red while in both cultivars it increased at Cut2. A salinity × cultivar interaction was observed mainly due to a change in scale between the two cultivars. Mg concentration was mostly affected by salinity and to a lesser extent by cultivar. The salinity effect was reflected solely on the High NaCl treatment which resulted in the highest Mg concentration, whereas no other salinity treatment differed from the Control. The effect of cut on Mg concentration was non-significant although it was implicated in a cultivar × cut interaction since Mg concentration in Green increased at Cut2 whereas in Red it was unaltered. Leaf sodium concentration was significantly affected by all the experimental factors examined. It was higher in Green and increased in response to all salinity treatments, except for the Moderate Mix treatment, which did not differ significantly from the non-salt Control treatment. The highest Na concentration was attained in response to the High NaCl treatment. The mean cultivar Na concentration doubled at Cut2 relative to Cut1, however, a cultivar × cut interaction revealed that the Na increase in Red was non-significant, whereas in Green a significant increase by 152.6% was noted. Chlorine concentration was significantly affected by salinity and cultivar but unlike sodium concentration, it demonstrated no significant change in response to the order of cuts. Chlorine concentration was higher in Red rather than Green and also incurred a significant increase with salinity treatments relative to the Control. The high salinity treatments induced higher Cl concentration than the moderate treatments while no significant differences were observed between the isomolar mix and plain NaCl treatments. None of the factors examined except salinity had a significant effect on the basil leaf nitrate concentration, where a tendency for reduced NO_3_ concentration was apparent with increasing salinity treatments (Table 5).

**TABLE 4:** Analysis of variance (ANOVA) and mean comparisons for mineral [nitrogen (N), phosphorus (P), sulfur (S), potassium (K), calcium (Ca), magnesium (Mg), sodium (Na), and chlorine (Cl)] concentration of Green and Red cultivars cultivated under iso-osmotic salinity treatments and harvested in two sequential cuts.

Source of variance	N	P	S	K	Ca	Mg	Na	Cl
	(g kg^–1^ DW)	(g kg^–1^ DW)	(g kg^–1^ DW)	(g kg^–1^ DW)	(g kg^–1^ DW)	(g kg^–1^ DW)	(g kg^–1^ DW)	(g kg^–1^ DW)
Cultivar (V)	***	*	***	***	***	*	***	**
Salinity (S)	ns	**	ns	ns	***	***	***	***
Cut (C)	ns	***	ns	**	***	ns	***	ns
S × V	ns	ns	ns	ns	**	ns	ns	ns
S × C	ns	ns	ns	ns	ns	ns	***	ns
V × C	ns	***	ns	ns	ns	**	**	ns
S × V × C	ns	**	ns	ns	ns	ns	ns	ns

	**Means ± standard error**							
	
**Cultivar**								
Green	45.99 ± 0.34a	10.45 ± 0.65a	1.06 ± 0.04b	53.84 ± 1.13a	7.61 ± 0.50b	2.77 ± 0.15b	3.46 ± 0.64a	20.24 ± 2.19b
Red	43.00 ± 0.43b	8.58 ± 0.44b	1.61 ± 0.07a	45.48 ± 1.65b	10.66 ± 0.77a	3.17 ± 0.18a	2.17 ± 0.47b	24.22 ± 2.81a
**Salinity**								
Control	45.09 ± 0.33	11.49 ± 0.39a	1.49 ± 0.12	48.73 ± 2.17	6.82 ± 0.39c	2.41 ± 0.14b	0.86 ± 0.13d	1.15 ± 0.10c
Moderate Mix	44.89 ± 0.75	11.19 ± 1.04a	1.41 ± 0.11	47.79 ± 2.96	10.03 ± 0.76b	2.42 ± 0.15b	1.15 ± 0.18cd	21.06 ± 1.10b
Moderate NaCl	44.66 ± 0.80	10.05 ± 0.71ab	1.38 ± 0.12	50.07 ± 2.25	7.18 ± 0.55c	3.06 ± 0.21b	2.77 ± 0.50b	19.29 ± 1.20b
High Mix	42.55 ± 0.88	7.24 ± 0.91b	1.20 ± 0.12	48.15 ± 2.81	14.16 ± 1.44a	2.86 ± 0.17b	2.23 ± 0.61bc	36.09 ± 2.29a
High NaCl	45.38 ± 0.80	7.44 ± 0.75b	1.15 ± 0.11	54.02 ± 2.36	8.09 ± 0.80bc	4.03 ± 0.27a	7.05 ± 1.17a	33.60 ± 2.25a
**Cut**								
Cut1	44.17 ± 0.47	10.20 ± 0.58a	1.40 ± 0.08	46.52 ± 1.56b	8.24 ± 0.61b	2.86 ± 0.17	1.93 ± 0.26b	22.44 ± 2.64
Cut2	45.10 ± 0.47	8.79 ± 0.56b	1.25 ± 0.07	53.33 ± 1.36a	9.99 ± 0.75a	3.06 ± 0.16	3.80 ± 0.76a	21.80 ± 2.39
**S × V**								
Control × Green	45.87 ± 0.40	11.66 ± 0.63	1.24 ± 0.08	52.58 ± 2.11	6.21 ± 0.46c	2.14 ± 0.17	0.98 ± 0.18	0.98 ± 0.10
Moderate Mix × Green	46.69 ± 0.72	13.67 ± 1.52	1.22 ± 0.09	51.21 ± 3.14	9.07 ± 0.97bc	2.38 ± 0.22	1.30 ± 0.29	19.43 ± 0.70
Moderate NaCl × Green	46.64 ± 0.65	11.66 ± 0.80	1.03 ± 0.06	54.44 ± 2.56	6.42 ± 0.84c	2.77 ± 0.15	3.72 ± 0.85	17.74 ± 1.22
High Mix × Green	44.26 ± 0.68	8.13 ± 1.64	0.94 ± 0.09	53.65 ± 2.30	10.65 ± 1.53b	2.77 ± 0.33	2.70 ± 1.06	33.39 ± 2.66
High NaCl × Green	46.17 ± 0.94	7.73 ± 1.20	0.89 ± 0.08	56.80 ± 2.51	6.43 ± 0.77c	3.63 ± 0.34	7.74 ± 1.67	30.19 ± 1.98
Control × Red	44.30 ± 0.28	11.31 ± 0.50	1.74 ± 0.18	44.87 ± 3.20	7.42 ± 0.57bc	2.67 ± 0.18	0.75 ± 0.20	1.36 ± 0.13
Moderate Mix × Red	43.08 ± 0.79	9.12 ± 0.75	1.60 ± 0.19	44.38 ± 4.90	10.99 ± 1.11b	2.46 ± 0.21	1.00 ± 0.22	22.69 ± 1.95
Moderate NaCl × Red	42.69 ± 0.92	8.45 ± 0.74	1.72 ± 0.11	45.70 ± 2.84	7.93 ± 0.64bc	3.35 ± 0.37	1.83 ± 0.10	20.84 ± 1.96
High Mix × Red	40.42 ± 1.04	6.35 ± 0.78	1.46 ± 0.14	42.64 ± 3.88	17.67 ± 0.90a	2.95 ± 0.14	1.76 ± 0.65	38.79 ± 3.58
High NaCl × Red	44.00 ± 1.35	7.03 ± 0.77	1.51 ± 0.12	50.13 ± 4.13	10.4 ± 0.84b	4.60 ± 0.31	6.08 ± 1.66	38.38 ± 3.93
**S × C**								
Control × Cut1	44.76 ± 0.41	11.29 ± 0.54	1.68 ± 0.21	45.94 ± 3.23	6.46 ± 0.57	2.26 ± 0.23	0.98 ± 0.21d	1.08 ± 0.17
Moderate Mix × Cut1	44.04 ± 0.97	12.41 ± 1.71	1.50 ± 0.13	43.66 ± 4.38	8.14 ± 0.56	2.37 ± 0.24	0.87 ± 0.15d	18.92 ± 0.50
Moderate NaCl × Cut1	44.75 ± 0.98	10.71 ± 1.00	1.33 ± 0.17	47.03 ± 2.65	6.65 ± 0.51	2.84 ± 0.16	2.20 ± 0.16bcd	16.67 ± 1.24
High Mix × Cut1	42.21 ± 1.24	8.23 ± 1.34	1.21 ± 0.15	45.38 ± 4.04	12.56 ± 1.97	2.64 ± 0.21	1.48 ± 0.57cd	35.67 ± 3.56
High NaCl × Cut1	45.10 ± 1.33	8.34 ± 1.02	1.29 ± 0.17	50.59 ± 3.51	7.38 ± 1.01	4.21 ± 0.37	4.09 ± 0.43b	36.29 ± 3.91
Control × Cut2	45.41 ± 0.52	11.69 ± 0.60	1.30 ± 0.07	51.51 ± 2.67	7.17 ± 0.56	2.55 ± 0.17	0.74 ± 0.17d	1.21 ± 0.12
Moderate Mix × Cut2	45.73 ± 1.11	9.72 ± 0.77	1.32 ± 0.19	51.93 ± 3.54	11.92 ± 0.90	2.47 ± 0.19	1.43 ± 0.29cd	23.21 ± 1.81
Moderate NaCl × Cut2	44.58 ± 1.36	9.40 ± 1.03	1.42 ± 0.19	53.11 ± 3.40	7.70 ± 0.99	3.29 ± 0.38	3.34 ± 0.96bc	21.91 ± 1.42
High Mix × Cut2	43.23 ± 1.09	5.76 ± 0.67	1.19 ± 0.22	52.31 ± 2.95	16.56 ± 1.63	3.19 ± 0.21	3.35 ± 1.11bcd	36.71 ± 2.70
High NaCl × Cut2	45.73 ± 0.91	6.53 ± 1.05	1.01 ± 0.15	57.45 ± 2.73	8.79 ± 1.27	3.86 ± 0.40	10.01 ± 1.53a	30.90 ± 2.04
**V × C**								
Green × Cut1	45.61 ± 0.47	11.90 ± 0.80a	1.12 ± 0.07	50.38 ± 1.23	6.41 ± 0.34	2.42 ± 0.18b	1.96 ± 0.40b	19.45 ± 3.15
Green × Cut2	46.37 ± 0.49	8.90 ± 0.88b	1.00 ± 0.06	57.30 ± 1.44	8.82 ± 0.84	3.11 ± 0.20a	4.95 ± 1.11a	21.03 ± 3.16
Red × Cut1	42.73 ± 0.64	8.49 ± 0.58b	1.68 ± 0.09	42.66 ± 2.54	10.07 ± 0.98	3.30 ± 0.24a	1.89 ± 0.36b	25.65 ± 4.26
Red × Cut2	43.38 ± 0.56	8.67 ± 0.70b	1.54 ± 0.09	48.75 ± 1.71	11.34 ± 1.22	3.01 ± 0.27a	2.48 ± 0.93b	22.68 ± 3.76

Salinity treatments: Control = non-salt; Moderate Mix = (12.5 mM NaCl + 8.3 mM CaCl_2_); Moderate NaCl = 25 mM NaCl; High Mix = (25 mM NaCl + 16.6 mM CaCl_2_); High NaCl = 50 mM NaCl. * Significant effect at the 0.05 level, ** 0.01 level, *** 0.001 level, ns = non-significant effect. Data represent means ± SE of 3 replicates (n = 3) consisting of 10 plants each. Treatment means within each column followed by different letters denote significant differences (P < 0.05) according to the Tukey–Kramer HSD test.

**TABLE 5:** Analysis of variance (ANOVA) and mean comparisons for nitrate concentration, ABTS antioxidant activity, total concentration of phenolic compounds, and ascorbic acid concentration of Green and Red cultivars grown hydroponically under iso-osmotic salinity treatments and harvested in two sequential cuts.

Source of variance	Nitrate (μg g^–1^ FW)	ABTS (g Trolox eq kg^–1^ DM)	Phenolics (mg gallic acid 100 g^–1^ DW)	Ascorbic acid (mg 100 g^–1^ FW)
Cultivar (V)	ns	ns	***	***
Salinity (S)	*	***	***	*
Cut (C)	ns	***	***	***
S × V	ns	ns	ns	ns
S × C	ns	ns	ns	ns
V × C	ns	ns	ns	***

	**Means ± standard error**			
	
**Cultivar**				
Green	2669 ± 136	9.03 ± 0.39	52.60 ± 3.30b	25.70 ± 1.70b
Red	2599 ± 147	12.81 ± 0.51	97.50 ± 2.90a	52.60 ± 4.60a
**Salinity**				
Control	3311 ± 150a	9.18 ± 1.02b	61.40 ± 7.20c	31.30 ± 5.10b
Moderate Mix	2891 ± 164b	11.51 ± 0.61a	75.80 ± 8.30ab	44.60 ± 9.00a
Moderate NaCl	2803 ± 174b	11.63 ± 1.05a	78.70 ± 8.90ab	41.70 ± 8.50a
High Mix	2093 ± 133c	11.99 ± 0.91a	87.50 ± 8.60a	44.80 ± 4.20a
High NaCl	1978 ± 220c	10.23 ± 0.71ab	72.00 ± 7.00bc	31.70 ± 4.50b
**Cut**				
Cut1	2753 ± 126	9.72 ± 0.47b	65.30 ± 4.80b	30.00 ± 2.30b
Cut2	2509 ± 153	12.03 ± 0.59a	84.80 ± 4.90a	48.20 ± 5.10a
**S × V**				
Control × Green	3133 ± 182	6.28 ± 0.26	39.40 ± 2.60	16.90 ± 1.80
Moderate Mix × Green	2841 ± 295	10.03 ± 0.70	50.40 ± 3.00	22.20 ± 2.80
Moderate NaCl × Green	3052 ± 246	9.65 ± 0.90	52.90 ± 7.60	32.90 ± 2.70
High Mix × Green	2170 ± 195	10.76 ± 0.63	65.00 ± 10.90	34.70 ± 3.90
High NaCl × Green	2150 ± 368	8.46 ± 0.42	55.20 ± 7.10	21.80 ± 1.70
Control × Red	3489 ± 231	12.09 ± 1.06	83.40 ± 5.30	45.70 ± 5.30
Moderate Mix × Red	2941 ± 174	12.99 ± 0.50	101.10 ± 6.20	67.10 ± 12.10
Moderate NaCl × Red	2553 ± 218	13.61 ± 1.58	104.40 ± 5.30	50.40 ± 16.70
High Mix × Red	2002 ± 190	13.48 ± 1.72	110.00 ± 2.00	54.90 ± 4.70
High NaCl × Red	1772 ± 212	12.00 ± 0.88	88.80 ± 7.20	43.50 ± 6.60
**S × C**				
Control × Cut1	3347 ± 213	8.66 ± 1.44	53.90 ± 9.00	25.30 ± 5.10
Moderate Mix × Cut1	3105 ± 185	11.37 ± 1.19	69.80 ± 11.10	29.10 ± 6.30
Moderate NaCl × Cut1	2850 ± 179	9.43 ± 0.70	66.00 ± 12.60	27.90 ± 3.40
High Mix × Cut1	2169 ± 206	10.04 ± 1.05	77.50 ± 13.30	39.90 ± 5.10
High NaCl × Cut1	2295 ± 319	9.14 ± 0.55	59.50 ± 8.50	27.70 ± 4.60
Control × Cut2	3276 ± 231	9.71 ± 1.55	68.90 ± 11.10	37.30 ± 8.60
Moderate Mix × Cut2	2677 ± 256	11.64 ± 0.43	81.70 ± 12.90	60.20 ± 14.80
Moderate NaCl × Cut2	2755 ± 316	13.84 ± 1.56	91.30 ± 11.30	55.50 ± 15.10
High Mix × Cut2	2003 ± 172	13.62 ± 1.07	97.50 ± 10.40	49.60 ± 6.60
High NaCl × Cut2	1597 ± 220	11.32 ± 1.19	84.40 ± 9.00	36.50 ± 8.40
**V × C**				
Green × Cut1	2894 ± 158	8.29 ± 0.47	41.90 ± 1.90	23.60 ± 2.90c
Green × Cut2	2445 ± 211	9.78 ± 0.58	63.20 ± 5.00	27.80 ± 1.80bc
Red × Cut1	2613 ± 196	11.24 ± 0.61	88.80 ± 3.90	36.40 ± 2.70bc
Red × Cut2	2583 ± 228	14.28 ± 0.61	106.30 ± 3.10	70.00 ± 6.40a

Salinity treatments: Control = non-salt; Moderate Mix = (12.5 mM NaCl + 8.3 mM CaCl_2_); Moderate NaCl = 25 mM NaCl; High Mix = (25 mM NaCl + 16.6 mM CaCl_2_); High NaCl = 50 mM NaCl. * Significant effect at the 0.05 level, *** 0.001 level, ns = non-significant effect. Data represent means ± standard error of 3 replicates (n = 3) consisting of 10 plants each. Treatment means within each column followed by different letters denote significant differences (p < 0.05) according to the Tukey–Kramer HSD test. Three-way interactions were either non-significant or had a very low contribution to the total variance of the examined variables.

### Quality and Functional Attributes

The ABTS antioxidant activity (ABTS) was significantly affected by salinity and cut. The ABTS was invariably increased by all salinity treatments compared to the control, except for the High NaCl treatment which was not significantly different from the Control. The ABTS was also significantly higher at Cut2 than Cut1. The total phenolic concentration was significantly affected by salinity, cultivar, and cut. Phenolics were higher in Red by 85.3% relative to Green and in both cultivars, the phenolic concentration increased at Cut2 by 29.9%. Moreover, all salinity treatments increased the leaf phenolic concentration except for the High NaCl treatment which did not differ significantly from the Control. The total AsA concentration was marginally affected by salinity (*p* ≤ 0.05) and more affected by cultivar and cut (*p* ≤ 0.05). Higher ascorbate concentration was found in Red, and though it increased at Cut2 in both cultivars, this increase was also more pronounced in Red.

### Volatile Aromatic Compounds Profile

A total of 84 VOCs were identified in the basil aromatic profile, accounting for 16 monoterpene hydrocarbons, 10 oxygenated monoterpenes, 18 sesquiterpene hydrocarbons, 11 aliphatic alcohols, 8 aliphatic aldehydes, 11 aliphatic esters, 2 aliphatic ketones, and 8 aromatic compounds (Supplementary Table 1). Among these, seven major VOCs were identified in the two cultivars, which contributed individually ≥2% and collectively near 90% to the total VOCs relative abundance (Table 6). These included two monoterpene hydrocarbons: β-Myrcene (woody, vegetative, citrus fruity, and minty nuance) and (Z)-β-Ocimene (sweet herbal); two oxygenated monoterpenes: eucalyptol (fresh mint, spicy, cooling) and β-Linalool (floral, woody, sweet, spicy, tropical); one aliphatic aldehyde: (E)-2-hexenal (green, fruity, fresh, herbal, citrus); one aliphatic alcohol: 1-octen-3-ol (mushroom, earthy, fungal, green, oily, vegetative, umami sensation, and savory-brothy); and one sesquiterpene hydrocarbon: *trans-*α-bergamotene (citrusy, fresh, earthy green odor).

**TABLE 6:** Analysis of variance (ANOVA) and mean comparisons for the relative abundance (%) of major aroma volatile components [β-myrcene, eucalyptol, (E)-2-hexenal, (Z)-β-ocimene, 1-octen-3-ol, β-linalool, and *trans-*α-bergamotene] in Green and Red cultivars grown hydroponically under iso-osmotic salinity treatments and harvested in two sequential cuts.

Source of variance	β-myrcene	Eucalyptol	(E)-2-hexenal	(Z)-β-ocimene	l-Octen-3-ol	β-linalool	*Trans-*α-bergamotene
Cultivar (V)	*	ns	*	***	**	***	***
Salinity (S)	ns	ns	ns	*	***	***	ns
Cut (C)	ns	*	**	***	ns	ns	ns
S × V	ns	*	ns	*	*	ns	ns
S × C	**	ns	ns	*	ns	*	ns
V × C	***	***	ns	***	ns	***	***
S × V × C	*	**	ns	**	ns	ns	*

	**Mean percentage fraction of total volatile area ± standard error**						
	
**Cultivar**							
Green	4.86 ± 0.15b	31.52 ± 1.02	2.17 ± 0.12b	4.80 ± 0.47a	5.51 ± 0.13a	19.5 ± 0.45b	10.17 ± 0.54a
Red	5.40 ± 0.28a	32.91 ± 0.73	2.67 ± 0.16a	1.48 ± 0.05b	5.02 ± 0.24b	24.3 ± 0.47a	6.45 ± 0.43b
**Salinity**							
Control	4.76 ± 0.26	31.16 ± 1.63	2.26 ± 0.17	3.56 ± 0.69a	6.01 ± 0.24a	23.17 ± 0.99a	8.23 ± 1.25
Moderate Mix	5.14 ± 0.29	32.84 ± 0.84	2.57 ± 0.27	3.55 ± 0.63a	5.42 ± 0.19ab	22.84 ± 0.76a	7.02 ± 0.60
Moderate NaCl	4.83 ± 0.36	31.92 ± 1.54	2.31 ± 0.19	3.13 ± 0.70ab	5.86 ± 0.20a	22.09 ± 0.93ab	8.23 ± 0.77
High Mix	5.33 ± 0.34	31.67 ± 1.17	2.41 ± 0.33	3.37 ± 0.95ab	4.28 ± 0.31c	20.17 ± 1.00b	9.95 ± 1.15
High NaCl	5.60 ± 0.50	33.42 ± 1.87	2.50 ± 0.19	2.32 ± 0.74b	4.66 ± 0.28bc	20.58 ± 1.22b	8.59 ± 0.80
**Cut**							
Cut1	5.22 ± 0.27	33.46 ± 0.91a	2.67 ± 0.13a	2.48 ± 0.40b	5.39 ± 0.18	21.69 ± 0.53	8.12 ± 0.50
Cut2	5.02 ± 0.17	30.92 ± 0.83b	2.14 ± 0.15b	3.91 ± 0.49a	5.15 ± 0.21	21.94 ± 0.75	8.63 ± 0.69
**S × V**							
Control × Green	4.77 ± 0.28	27.70 ± 2.07	2.14 ± 0.12	5.78 ± 0.29a	5.69 ± 0.33ab	20.83 ± 1.26	11.24 ± 1.65
Moderate Mix × Green	4.69 ± 0.24	32.96 ± 1.58	2.11 ± 0.28	5.56 ± 0.36a	5.63 ± 0.14ab	21.06 ± 0.56	8.46 ± 0.46
Moderate NaCl × Green	4.28 ± 0.42	32.38 ± 2.70	2.21 ± 0.30	4.79 ± 1.03ab	6.03 ± 0.30ab	20.03 ± 0.94	8.86 ± 1.26
High Mix × Green	5.30 ± 0.30	30.31 ± 1.57	1.81 ± 0.36	4.80 ± 1.55ab	4.95 ± 0.30bc	17.64 ± 0.70	12.58 ± 0.88
High NaCl × Green	5.24 ± 0.33	34.26 ± 2.85	2.56 ± 0.18	3.07 ± 1.32bc	5.25 ± 0.26abc	17.96 ± 0.71	9.71 ± 0.93
Control × Red	4.75 ± 0.47	34.63 ± 1.62	2.37 ± 0.34	1.33 ± 0.08c	6.34 ± 0.30a	25.52 ± 0.70	5.22 ± 0.75
Moderate Mix-Red	5.59 ± 0.48	32.72 ± 0.75	3.03 ± 0.41	1.55 ± 0.07c	5.21 ± 0.36abc	24.63 ± 0.96	5.58 ± 0.74
Moderate NaCl × Red	5.37 ± 0.51	31.46 ± 1.73	2.41 ± 0.25	1.48 ± 0.10c	5.70 ± 0.28ab	24.14 ± 1.13	7.60 ± 0.91
High Mix × Red	5.36 ± 0.71	33.31 ± 1.59	3.13 ± 0.41	1.65 ± 0.18c	3.46 ± 0.32d	23.20 ± 0.77	6.80 ± 1.24
High NaCl × Red	6.02 ± 1.06	32.41 ± 2.56	2.42 ± 0.39	1.42 ± 0.09c	3.95 ± 0.33cd	23.73 ± 1.71	7.25 ± 1.18
**S × C**							
Control × Cut1	4.05 ± 0.21b	34.09 ± 2.16	2.09 ± 0.25	3.41 ± 1.03a	5.98 ± 0.23	24.82 ± 0.97a	7.19 ± 1.00
Moderate Mix × Cut1	5.44 ± 0.46ab	32.98 ± 1.36	2.81 ± 0.26	3.65 ± 0.90a	5.63 ± 0.34	22.47 ± 0.92	6.96 ± 0.78
Moderate NaCl × Cut1	5.08 ± 0.64ab	33.07 ± 2.37	2.56 ± 0.23	2.38 ± 0.75ab	5.78 ± 0.33	21.32 ± 0.87bc	8.21 ± 0.78
High Mix × Cut1	5.64 ± 0.45ab	32.48 ± 1.25	3.01 ± 0.38	1.97 ± 0.89ab	4.42 ± 0.52	20.40 ± 0.77bc	9.44 ± 1.87
High NaCl × Cut1	6.02 ± 1.00a	34.91 ± 3.49	2.94 ± 0.23	0.72 ± 0.37b	5.11 ± 0.30	19.01 ± 1.00c	8.92 ± 0.65
Control × Cut2	5.48 ± 0.23ab	28.24 ± 1.91	2.42 ± 0.25	3.70 ± 1.00a	6.04 ± 0.44	21.52 ± 1.49abc	9.27 ± 2.34
Moderate Mix × Cut2	4.84 ± 0.34ab	32.69 ± 1.11	2.33 ± 0.49	3.45 ± 0.96a	5.21 ± 0.17	23.21 ± 1.27ab	7.08 ± 0.99
Moderate NaCl × Cut2	4.57 ± 0.35ab	30.77 ± 2.07	2.07 ± 0.29	3.89 ± 1.17a	5.94 ± 0.26	22.86 ± 1.69ab	8.25 ± 1.40
High Mix × Cut2	4.96 ± 0.52ab	30.70 ± 2.17	1.69 ± 0.38	5.05 ± 1.59a	4.10 ± 0.34	19.89 ± 2.14bc	10.57 ± 1.35
High NaCl × Cut2	5.24 ± 0.43ab	32.18 ± 2.01	2.13 ± 0.19	3.66 ± 1.06a	4.28 ± 0.41	21.89 ± 2.02abc	8.32 ± 1.42
**V × C**							
Green × Cut1	4.45 ± 0.15c	35.18 ± 1.22a	2.50 ± 0.11	3.29 ± 0.72b	5.79 ± 0.16	20.51 ± 0.68c	8.89 ± 0.74bc
Green × Cut2	5.27 ± 0.22ab	27.87 ± 0.94c	1.84 ± 0.17	6.31 ± 0.25a	5.22 ± 0.19	18.50 ± 0.48c	11.46 ± 0.66a
Red × Cut1	6.04 ± 0.44ab	31.61 ± 1.22b	2.86 ± 0.24	1.62 ± 0.08c	4.96 ± 0.31	22.96 ± 0.68b	7.03 ± 0.64bc
Red × Cut2	4.76 ± 0.24bc	34.20 ± 0.68ab	2.47 ± 0.21	1.35 ± 0.03c	5.07 ± 0.38	25.63 ± 0.44a	5.61 ± 0.52c

Salinity treatments: Control = non-salt; Moderate Mix = (12.5 mM NaCl + 8.3 mM CaCl_2_); Moderate NaCl = 25 mM NaCl; High Mix = (25 mM NaCl + 16.6 mM CaCl_2_); High NaCl = 50 mM NaCl. * Significant effect at the 0.05 level, ** 0.01 level, *** 0.001 level, ns = non-significant effect. Data represent means ± SE of 3 replicates (n = 3) consisting of 10 plants each. Treatment means within each column followed by different letters denote significant differences (p < 0.05) according to the Tukey–Kramer HSD test.

The relative abundance of β-Myrcene was affected only by a cultivar, being higher in red basil. Eucalyptol was only affected by the cut sequence, and it was subject to significant cultivar × cut interaction as in Green, it increased at Cut2 whereas in Red it did not change significantly. Salinity did not have a significant effect on (E)-2-hexenal as opposed to cultivar and cut, being more abundant in Red rather than Green, and in Cut1 compared to Cut2. The relative abundance of (Z)-β-ocimene was subject to significant interactions involving the three main factors of the model examined. (Z)-β-ocimene was more abundant in Green irrespective of salinity treatment and in this cultivar, it nearly doubled from Cut1 to Cut2, as opposed to Red wherein no difference was observed between cuts. Salinity had no effect on the relative abundance of this component in Red, however, in Green, it increased significantly in the High NaCl treatment compared to the Control and Moderate Mix treatments. The relative abundance of 1-octen-3-ol was higher in Green than Red; however, this difference was apparent only in response to the High Mix and High NaCl treatments. β-linalool was relatively more abundant in Red in which also increased at Cut2, whereas in Green it did not change in response to cut order. In both cultivars, the presence of β-linalool decreased in the High Mix and High NaCl treatments. *Trans-*α-bergamotene incurred a significant effect from the cultivar as it was overall more abundant in Green than Red; however, its relative abundance in Green increased significantly from Cut1 to Cut2, whereas in Red, no change was observed.

### Principal Component Analysis

**FIGURE 1:**
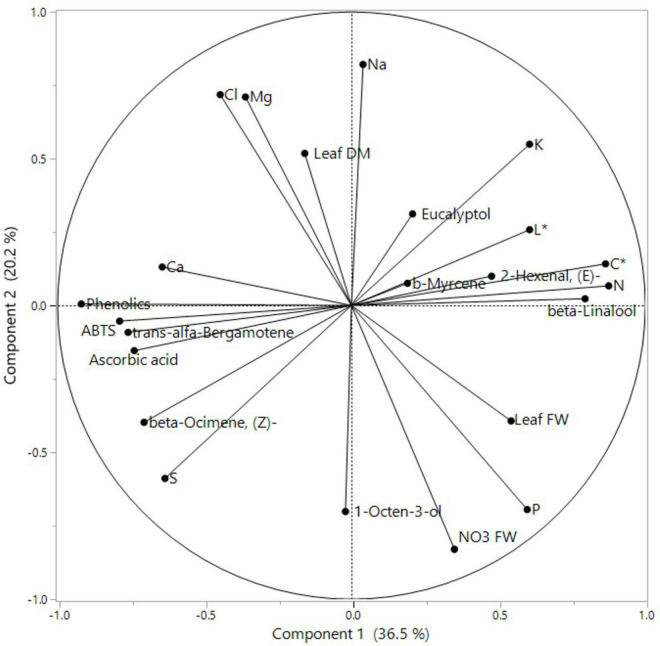
Principal component loading plot of principal component analysis (PCA) of morphometric and yield parameters, nutritional quality, aromatic profile, leaf colorimetry, and macronutrient concentration of Green and Red cultivars grown hydroponically under iso-osmotic salinity treatments and harvested in two sequential cuts. Salinity treatments: Control = non-salt; Moderate Mix = (12.5 mM NaCl + 8.3 mM CaCl_2_); Moderate NaCl = 25 mM NaCl; High Mix = (25 mM NaCl + 16.6 mM CaCl_2_); High NaCl = 50 mM NaCl.

**FIGURE 2:**
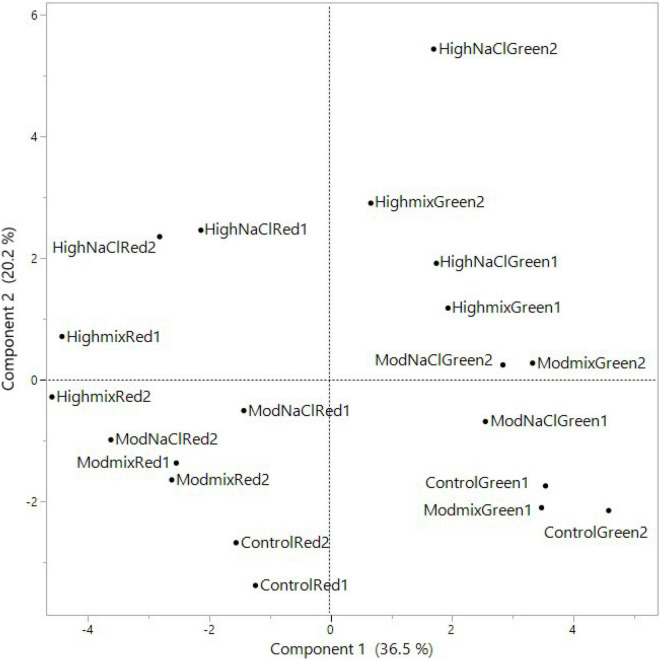
Scores of PCA of morphometric and yield parameters, nutritional quality, aromatic profile, leaf colorimetry and macronutrient concentration of Green and Red cultivars grown hydroponically under iso-osmotic salinity treatments and harvested in two sequential cuts. Salinity treatments: Control = non-salt; Moderate Mix = (12.5 mM NaCl + 8.3 mM CaCl_2_); Moderate NaCl = 25 mM NaCl; High Mix = (25 mM NaCl + 16.6 mM CaCl_2_); High NaCl = 50 mM NaCl.

A PCA was performed on all basil-analyzed data in relation to the three experimental factors: cultivars, iso-osmotic salinity concentrations, and sequential harvests. The loading plot and scores are reported in Figures 1, 2. PC1 was positively correlated with leaf FW, color components L* and C*, N, P, K, and beta-Linalool while it was negatively correlated with S, Ca, ABTS, phenolics, AsA concentrations but also with the relative percentage of beta-Ocimene, (Z)-, 2-Hexenal, (E)-, and *trans-*alfa-Bergamotene (Figure 1). Moreover, PC2 was positively correlated with leaf dry matter, K, Mg, Na, and Cl concentration while it was negatively correlated with P, S, and NO_3_ FW. The two basil cultivars were well separated but not uniformly clustered with respect to PC1 and PC2 (Figure 2). The green basil treatments from cuts 1 and 2 were distributed on the positive side of PC1 between upper and lower right quadrants, while the red basil cuts 1 and 2 treatments were distributed on the negative side of PC1, in the upper and lower left quadrants. The successive red basil cuts grown under the same salinity level were detected in the same quadrant, apart from the red basil cuts grown under high mix salinity. Similar behavior was observed for the two cuts of green basil except for green basil cuts grown under the moderate mix and moderate NaCl salinity treatments. Furthermore, the control treatments were in the negative quadrant of PC2 irrespective of cultivar type or cut. Quadrant 1 of the score plot included treatments that were characterized by a higher concentration of Na, K, or N while the treatments in quadrant 2 had the highest concentration of Cl, Mg, and phenolics. The lower negative side of PC1 (quadrant 3) included treatment combinations that produced plants characterized by high S and AsA concentration, increased antioxidant activity but also by a higher relative abundance (%) of the major aroma volatile components like *trans-*α-bergamotene, (Z)-β-ocimene, and 1-Octen-3-ol. Treatments in the lower right quadrant (quadrant 4) produced leaves with the highest FW but they were also characterized by a higher concentration of P and nitrates.

## Discussion

### Salinity Affects Morphometric and Yields Parameters in a Cultivar-Dependent Manner

In the present work, the Red and Green basil cultivars showed different responses to moderate and high salt stress induced by NaCl and the mixture of NaCl and CaCl_2_ at isomolar concentrations. Relative to the weight and leaf area, Red appeared more tolerant to salinity than Green (Table 2). FW and leaf area in Green were significantly reduced by Moderate NaCl, High Mix, and High NaCl treatments, compared to the Control. On the contrary, Red showed no difference between treatments for these parameters, similar to the findings of Cruz et al. (2020) on red basil cultivars (Red basil, Dark Opal, and Basilico Rosso) exposed to high salinity levels. Bione et al. (2014) reported a linear decrease in the leaf FW of basil in response to increasing salinity levels and a strong genotypic effect on the adaptive capacity to the aforementioned abiotic stress. The reason why green cultivars showed a distinct response to saline treatments could be found in the high genetic variation of the *Ocimum* genus (Akbari et al., 2018). As previous studies suggested (Parida and Das, 2005; Bernstein et al., 2010), the reduction in fresh leaf weight observed in Green is derived primarily from the inhibitory effect of salt stress on leaf expansion. In fact, the decrease in Green fresh leaf weight under Moderate NaCl, High Mix, and High NaCl treatments was also associated with a reduction in leaf area.

Several authors (Attia et al., 2011; Ahmadi and Souri, 2018) suggest that the reduction in leaf weight is due to the inhibition of leaf initiation; however, in the present work, no significant changes were observed in the number of leaves per plant, corroborating that the reduction in leaf FW was due solely to the reduction in leaf area (Table 2). It should be noted that the more sensitive the Green cultivar, when not exposed to salt stress (Control), was characterized by the highest values for leaf area, fresh leaf weight, and DW, compared to the more tolerant Red cultivar. Nonetheless, at low salinity levels, the gap between the two cultivars narrowed, with Green linearly reducing DW, while Red did not follow the same trend. Specifically, compared to the Control, Red increased the DW with the Moderate Mix treatment. Although not significant, this increase could be due to the mitigating effect of calcium, which, as reported in the literature, could help plants preserve the integrity and functionality of membranes and cell walls by regulating the transport and ion exchange under NaCl salinity stress (Rengel, 1992; Borgognone et al., 2014a).

Chaparzadeh et al. (2004) suggested that high salinity may prompt growth reduction as an adaptive response to osmotic stress, which allows conservation of energy deployed to mitigate the risk of permanent damage. Such adaptations seem related to heat dissipation and transpiration adjustments mobilized under environmental stress conditions (Bridge et al., 2013). In light of the above, the unchanged leaf DM percentage contributes further evidence of Red’s higher tolerance to salinity. As argued by Bernstein et al. (2010), the ability of basil to maintain adequate tissue hydration is key to its salt stress tolerance. In fact, in the present work, Red did not significantly vary in leaf FW under saline treatments compared to the Control and, at the same time, it did not vary in leaf DM percentage, confirming the above statement. In the Red cultivar, the leaf water was unchanged, which indicates that Na and Cl ions were likely compartmentalized in the vacuoles (Tarchoune et al., 2013). By contrast, the Green cultivar probably also accumulated Na and Cl ions in the apoplast, leading to water loss through dehydration that affected the FW of the leaves (Attia et al., 2011). This suggests the likely occurrence of toxicity or ion imbalance in Green, which limited plant growth. Regarding the effect of cut sequence, except for leaf DM percentage, the two cultivars showed similar behavior in both cuts (Table 2). As argued by several authors, the increase in DW observed at the second cut could have resulted from greater root system growth and increased branching induced by the suppression of apical dominance (Zheljazkov et al., 2008; Nicoletto et al., 2013). Furthermore, the increase in cytokinins production in response to cut may have increased the number of leaf primordia in both cultivars and stimulated cell division leading to greater leaf area and fresh leaf weight (Wang et al., 2014).

### Leaf Colorimetry and Macronutrient Uptake Under Salt Stress: The Ameliorative Role of Calcium

Visual quality is a key driver for consumer choice of horticultural products, with coloration playing a determining role, especially for aromatic herbs (León et al., 2006). Color in plants is affected by various factors ranging from genetics to pre- and post-harvest factors (Pathare et al., 2013). In the current study, Chroma (C*) and Hue Angle (h°) in both basil cultivars were affected by the interaction between the investigated factors (Table 3). In agreement with the findings of Scagel et al. (2019), salt treatments did not change the colorimetric parameters in Green. In contrast, the leaf colorimetry of Red changed as salinity levels increased, corroborating analogous observations by Cruz et al. (2020). Red lost its distinctive pigmentation, showing a decrease in redness (a*), an increase in lightness (L*), and in yellowness (b*) in the Moderate NaCl, High Mix, and High NaCl treatments, compared to the Control (Table 3). Interestingly, plants in the Moderate Mix treatment did not vary in the above parameters compared to the Control, probably due to the mitigating effect of calcium and the low sodium levels (Table 4). However, this result was not observed in the High Mix treatment plants, where the sodium concentration was approximately 2.5 times higher than the Control (Table 4). Although the overall color intensity of Red increased at Cut2, this was probably due to the increase in yellowness (b*) rather than redness (a*), which decreased at Cut2. On the contrary, the Green cultivar’s colorimetry did not respond to cut sequence, which contrasts with the results reported by Ciriello et al. (2021b), possibly reflecting differences in the genetic materials used, the cultivation systems applied, and in growing seasons. The present results highlight how red-colored genotypes, despite being overall more tolerant to salinity and sequential cut stress than green cultivars, are also more prone to leaf color downgrading.

Apart from salinity and cut, the mineral profile of basil in the current study showed strong cultivar dependence (Table 4). The higher concentration of macroelements N, P, and K in Green, crucial to maintaining plant biological functions, may justify its higher yield compared to Red, which was characterized by a higher concentration of Ca, Mg, and less Na (Tables 2, 4; Karthika et al., 2018). The lower accumulation of Na in the leaves of Red probably relates to a desalination mechanism that transports this cation to the hypogeal apparatus (Attia et al., 2009). This likely mechanism activated by Red could underlie its greater tolerance to salt stress compared to Green (Table 2). Relative to the effect of cut, in agreement with Nicoletto et al. (2013) and Carillo et al. (2020), the second cut increased calcium and potassium concentration (Table 4). These elements play an essential role in promoting human health, such as stabilizing blood pressure, detoxification, and improving bone structure (Gharibzahedi and Jafari, 2017). Although an increase in FW was observed from the first to the second cut (Table 2), this was not associated with an increase in the leaf concentration of P and N. This could be attributed, as hypothesized for basil by Nicoletto et al. (2013), to the variation in environmental parameters during the two successive harvests. Although the detrimental impact of NaCl salinity on K uptake in different vegetables is known in the literature (Borgognone et al., 2014a), our results do not show significant differences in the concentration of this cation in leaves. As suggested by Scagel et al. (2019), this inconsistency can be explained by the fact that leaf nutrient concentration was determined in our study and not total plant uptake. Considering the reduction in leaf area, leaf FW, and DW in High Mix and High NaCl treatments (Table 2), it can be assumed that salinity reduced K uptake without altering leaf concentration. This hypothesis is confirmed by several studies on basil in which NaCl salinity, in addition to reducing fresh production, increased K concentration in leaves (Zuccarini and Okurowska, 2008; Attia et al., 2009; Scagel et al., 2017).

The addition of CaCl_2_ in the Moderate Mix and High Mix solutions evidently increased Ca and decreased the Na concentration compared to the equivalent isomolar Moderate NaCl and High NaCl solutions (Table 4). What was subsequently observed was not surprising since calcium is known to mitigate NaCl salinity by reducing the efficiency of leaf sodium uptake *via* the reduction in the permeability of the plasmalemma to this toxic cation and the increase in the K/Na ratio that enhances tolerance to salinity (Nedjimi and Daoud, 2009; Colla et al., 2013b). However, the beneficial effect of calcium in NaCl saline treatments was only observed in the moderate salinity treatment (Moderate Mix) where leaf area, leaf FW, and DW were unchanged compared to the non-salinized Control. In the high salinity treatment (High Mix), a strong reduction was observed in the above parameters as calcium was unable to mitigate the detrimental effects of salinity, probably due to the exceedingly high concentration of Cl that reached toxic levels even for Cl-tolerant plants (Scagel et al., 2017). Not least, the reduction in FW recorded in the High Mix versus the High NaCl treatments could be attributable not only to an increase in Cl but also to a decrease in P (Table 4). This element is a limiting factor for plant development as its deficiency leads to a reduction in photosynthesis and the ATP/ADP ratio (Formisano et al., 2021b).

### Moderate Salinity Improves Nutritional Quality Without Affecting the Aromatic Profile of Basil

In our study, leaf nitrate concentration was influenced only by saline treatments (Table 5). Nitrate decreased significantly in plants treated with moderate and high salt levels (Moderate Mix, Moderate NaCl, High Mix, and High NaCl) compared to the Control, with the lowest values obtained in high-salinity treatments. Salinity depresses the rate of nitrate transport through xylem vessels, leading to reduced growth (Borgognone et al., 2014a), as evidenced in our results (Table 1). The reduction in nitrate was due to the increase in Cl concentration, which showed the opposite trend (Table 4). In fact, chlorine, due to its recognized antagonism to nitrate, results in a downregulation of its net uptake (Carillo et al., 2020). Several studies have shown that plant tolerance to high salinity levels is a function of the ability to implement active defense mechanisms such as the production of antioxidant compounds (Rouphael and Kyriacou, 2018; Carillo et al., 2020; Farsaraei et al., 2020). Indeed, in our study, the most salinity-tolerant Red cultivar had higher concentrations of AsA and total phenols (Table 5). The higher concentration of total phenols in the leaves of Red can be attributed to the constitutive presence of anthocyanins, belonging to the flavonoid class, which confer the characteristic red pigmentation. Regardless of the cultivar and cut, salinity significantly altered the polyphenol concentration (Table 5). Consistent with several studies (Petropoulos et al., 2017), moderate salinity levels (Moderate Mix and Moderate NaCl) increased polyphenols without affecting yield, compared to the Control (Table 2), achieving the desired compromise between yield and increase in beneficial secondary metabolites. The opposite trend was observed with high salinity levels. In particular, the High NaCl treatment reduced yield without increasing phenolic concentration and ABTS antioxidant activity, thus demonstrating the strong dependence of the synthesis of antioxidant compounds on the magnitude of salt stress (Was̈kiewicz et al., 2013). In contrast, the application of calcium chloride in the high salinity treatment (High Mix), although it reduced yield compared to the Control, resulted in a significant increase in total phenols. Indeed, Ca ion is a crucial ubiquitous messenger involved in plant biosignal transduction (Anil and Sankara Rao, 2001) that can influence physiological activities such as growth and development but especially resistance and adaptation to stressors, partly through the biosynthesis of phenols, known metabolites involved in stress response (Ma et al., 2019). The role of phenols as “stress mitigants” is confirmed by their increase after the cut (Table 5), as observed by several authors (Nicoletto et al., 2013; Ciriello et al., 2021b) in basil. The increase in total phenols could be attributable to the upregulation of the Phenylalanine-ammonia-lyase (PAL) enzyme in response to the mechanical stress induced by the cut (Ciriello et al., 2021b). Additionally, the improved production performance at the second cut (Table 2) may have resulted from increased photosynthate allocation to the shikimate metabolic pathway (Shaw et al., 1998; Crozier et al., 2007). In our study, the second cut increased ABTS antioxidant activity, contrary to the findings of Corrado et al. (2020a), while it promoted vitamin C biosynthesis exclusively in the Red cultivar (Table 5). This observation highlights how genetic material may be implicated in plant response to cut stress.

The gas chromatography/mass spectrometry (GC/MS) analysis showed that the aroma profile of Red and Green basil was predominantly characterized by eucalyptol (Table 6), in contrast to several studies in which β-linalool was the most abundant compound (Marotti et al., 1996; Ciriello et al., 2021a,b). This variation in the volatile composition may derive from the interaction of pre-harvest factors such as cultivar, environmental conditions, and cultural practices (Farsaraei et al., 2020). Similarly, abiotic stresses, such as salinity, can alter the volatilome in a manner highly dependent on the duration and magnitude of stress and the cultivar-specific tolerance (Niinemets, 2010). In our work, high salinity levels (High Mix and High NaCl) reduced β-linalool, probably due to the depression of carbon assimilation required to produce VOCs, as inferred from the reduced DW (Table 2). As is known in the literature, mutable conditions after the cut had a greater influence on the quality of the aroma profile (Table 6; Zheljazkov et al., 2008; Corrado et al., 2020a; Ciriello et al., 2021a,b). Specifically, the two basil cultivars responded differentially to the successive cuts, with an increase in β-myrcene and β-ocimene and a reduction in eucalyptol observed in Green, while an increase in β-linalool was observed in Red. Different growth conditions and mechanical stress signaling may have altered the metabolic pathways involving mono-oxygenated terpenes that share the same precursor (i.e., geranyl pyrophosphate; GPP). However, the singular increase in β-linalool found in Red, despite being in line with Ciriello et al. (2021b), confirms once more that gene expression of critical enzymes in the biosynthesis of oxygenated monoterpenes is strongly controlled by the interaction of genotype and stress in a way that is not yet completely clear (Chang et al., 2007).

## Conclusion

In the next decades, groundwater and soil salinization will pose a major challenge for growers in the Mediterranean coastal region. Understanding salinity thresholds could help growers select targeted crops capable of optimizing water and soil resources use to achieve high yields of premium quality vegetables. However, moderate salt stress can trigger adaptive mechanisms in plants leading to the production of the desired secondary metabolites. Our study showed that moderate salinity levels (Moderate Mix and Moderate NaCl) and high salinity levels in combination with calcium chloride (High Mix) improved the nutritional quality of the two differentially pigmented hydroponic basil genotypes by boosting phenol concentration and lowering nitrate concentration, without affecting the eucalyptol. The response of productive traits to salinity was not uniform among cultivars. Despite being more productive overall, the Green cultivar showed a linear decrease in yield as the salinity level increased. Although the Red cultivar did not show yield reduction in response to salinity, induced stress resulted in a drastic alteration of the typical pigmentation of the leaves that turned green. However, the addition of calcium to the moderate salt solution (Moderate Mix) reduced the detrimental effects mentioned above for both Red and Green cultivars, underlining its mitigating role against salt stress.

## Data Availability Statement

The raw data supporting the conclusions of this article will be made available by the authors, without undue reservation.

## Author Contributions

YR: conceptualization, project administration, resources, visualization, and supervision. MC, LF, MK, and YR: methodology, validation, formal analysis, investigation, and writing – original draft preparation. GS and AK: software and data curation. MC, LF, GS, AK, SD, MK, and YR: writing – review and editing. SD and YR: funding acquisition. All authors contributed to the article and approved the submitted version.

## Conflict of Interest

The authors declare that the research was conducted in the absence of any commercial or financial relationships that could be construed as a potential conflict of interest.

## Publisher’s Note

All claims expressed in this article are solely those of the authors and do not necessarily represent those of their affiliated organizations, or those of the publisher, the editors and the reviewers. Any product that may be evaluated in this article, or claim that may be made by its manufacturer, is not guaranteed or endorsed by the publisher.

## References

[B1] Ahmadi M. Souri M. K. (2018). Growth and mineral content of coriander (*Coriandrum sativum L*.) plants under mild salinity with different salts. *Acta Physiol. Plant.* 40 1–8. 10.1007/s11738-018-2773-x

[B2] Ahmed A. F. Attia F. A. K. Liu Z. Li C. Wei J. Kang W. (2019). Antioxidant activity and total phenolic content of essential oils and extracts of sweet basil (*Ocimum basilicum L*.) plants. *Food Sci. Hum. Wellness* 8 299–305. 10.1016/j.fshw.2019.07.004

[B3] Akbari G. A. Soltani E. Binesh S. Amini F. (2018). Cold tolerance, productivity and phytochemical diversity in sweet basil (*Ocimum basilicum L.)* accessions. *Ind. Crops Prod.* 124 677–684. 10.1016/j.indcrop.2018.08.048

[B4] Amor G. Sabbah M. Caputo L. Idbella M. De Feo V. Porta R. (2021). Basil essential oil: composition, antimicrobial properties, and microencapsulation to produce active chitosan films for food packaging. *Foods* 10:121. 10.3390/foods10010121 33430030 PMC7827191

[B5] Anil V. S. Sankara Rao K. (2001). Calcium-mediated signal transduction in plants: a perspective on the role of Ca2+ and CDPKs during early plant development. *J. Plant Physiol.* 158 1237–1256. 10.1078/0176-1617-00550

[B6] Attia H. Karray N. Ellili A. Msilini N. Lachaâl M. (2009). Sodium transport in basil. *Acta Physiol. Plant.* 31 1045–1051. 10.1007/s11738-009-0324-1

[B7] Attia H. Ouhibi C. Ellili A. Msilini N. Bouzaïen G. Karray N. (2011). Analysis of salinity effects on basil leaf surface area, photosynthetic activity, and growth. *Acta Physiol. Plant.* 33 823–833. 10.1007/s11738-010-0607-6

[B8] Barbieri G. Vallone S. Orsini F. Paradiso R. De Pascale S. Negre-Zakharov F. (2012). Stomatal density and metabolic determinants mediate salt stress adaptation and water use efficiency in basil (*Ocimum basilicum L*.). *J. Plant Physiol.* 169 1737–1746. 10.1016/j.jplph.2012.07.001 22840325

[B9] Bauer G. Schulze E. D. Mund M. (1997). Nutrient contents and concentrations in relation to growth of *Picea abies* and *Fagus sylvatica* along a European transect. *Tree Physiol.* 17 777–786. 10.1093/treephys/17.12.777 14759887

[B10] Bernstein N. Kravchik M. Dudai N. (2010). Salinity-induced changes in essential oil, pigments and salts accumulation in sweet basil (Ocimum basilicum) in relation to alterations of morphological development. *Ann. Appl. Biol.* 156 167–177. 10.1111/j.1744-7348.2009.00376.x

[B11] Bione M. A. A. Paz V. P. da Silva F. Ribas R. F. Soares T. M. (2014). Growth and production of basil in NFT hydroponic system under salinity. *Rev. Bras. Eng. Agric. e Ambient* 18 1228–1234. 10.1590/1807-1929/agriambi.v18n12p1228-1234

[B12] Bonilla I. El-Hamdaoui A. Bolaños L. (2004). Boron and calcium increase *Pisum sativum* seed germination and seedling development under salt stress. *Plant Soil* 267 97–107. 10.1007/s11104-005-4689-7

[B13] Borghesi E. Carmassi G. Uguccioni M. C. Vernieri P. Malorgio F. (2013). Effects of calcium and salinity stress on quality of lettuce in soilless culture. *J. Plant Nutr.* 36 677–690. 10.1080/01904167.2012.721909

[B14] Borgognone D. Cardarelli M. Lucini L. Colla G. (2014a). Does CaCl_2_ play a role in improving biomass yield and quality of cardoon grown in a floating system under saline conditions? *Hortscience* 49 1523–1528. 10.21273/hortsci.49.12.1523

[B15] Borgognone D. Cardarelli M. Rea E. Lucini L. Colla G. (2014b). Salinity source-induced changes in yield, mineral composition, phenolic acids and flavonoids in leaves of artichoke and cardoon grown in floating system. *J. Sci. Food Agric.* 94 1231–1237. 10.1002/jsfa.6403 24105819

[B16] Borgognone D. Rouphael Y. Cardarelli M. Lucini L. Colla G. (2016). Changes in biomass, mineral composition, and quality of cardoon in response to NO3–:Cl– ratio and nitrate deprivation from the nutrient solution. *Front. Plant Sci.* 7:978. 10.3389/fpls.2016.00978 27446196 PMC4928370

[B17] Bremner J. M. (1965). “Total nitrogen,” in *Methods of Soil Analysis. Part 2. Chemical And Microbiological Properties. Agronomy Monograph 9*, eds Black C. A. Evans D. Ensminger J. L. Clark F. E. (Madison, WI: American Society of Agronomy, Soil Science Society of America), 1149–1178.

[B18] Bridge L. J. Franklin K. A. Homer M. E. (2013). Impact of plant shoot architecture on leaf cooling: a coupled heat and mass transfer model. *J. R. Soc. Interface* 10:20130326. 10.1098/rsif.2013.0326 23720538 PMC4043166

[B19] Carillo P. Giordano M. Raimondi G. Napolitano F. Stasio E. Di Kyriacou M. C. (2020). Physiological and nutraceutical quality of green and red pigmented lettuce in response to NaCl concentration in two successive harvests. *Agronomy* 10:1358. 10.3390/agronomy10091358

[B20] Ch M. Naz S. Sharif A. Akram M. Saeed M. (2015). Biological and pharmacological properties of the sweet basil (*Ocimum basilicum)*. *Br. J. Pharm. Res.* 7 330–339. 10.9734/bjpr/2015/16505

[B21] Chang X. Alderson P. G. Hollowood T. A. Hewson L. Wright C. J. (2007). Flavour and aroma of fresh basil are affected by temperature. *J. Sci. Food Agric.* 87 1381–1385. 10.1002/jsfa.2869

[B22] Chaparzadeh N. D’Amico M. L. Khavari-Nejad R. A. Izzo R. Navari-Izzo F. (2004). Antioxidative responses of calendula officinalis under salinity conditions. *Plant Physiol. Biochem.* 42 695–701. 10.1016/j.plaphy.2004.07.001 15474374

[B23] Cheruth A. J. Ramadhan K. I. M. Kurup S. S. (2016). Calcium supplementation ameliorates salinity stress in Lactuca sativa plants. *J. Appl. Hortic.* 18 138–140. 10.37855/jah.2016.v18i02.24

[B24] Ciriello M. Formisano L. El-Nakhel C. Corrado G. Pannico A. De Pascale S. (2021a). Morpho-Physiological responses and secondary metabolites modulation by preharvest factors of three hydroponically grown genovese basil cultivars. *Front. Plant Sci.* 12:619. 10.3389/fpls.2021.671026 33981328 PMC8107287

[B25] Ciriello M. Formisano L. El-Nakhel C. Kyriacou M. C. Soteriou G. A. Pizzolongo F. (2021b). Genotype and successive harvests interaction affects phenolic acids and aroma profile of genovese basil for pesto sauce production. *Foods* 10:278. 10.3390/foods10020278 33573127 PMC7911349

[B26] Ciriello M. Pannico A. El-Nakhel C. Formisano L. Cristofano F. Duri L. G. (2020). Sweet basil functional quality as shaped by genotype and macronutrient concentration reciprocal action. *Plants* 9:1786. 10.3390/plants9121786 33339286 PMC7767113

[B27] Colla G. Rouphael Y. Cardarelli M. Svecova E. Rea E. Lucini L. (2013a). Effects of saline stress on mineral composition, phenolic acids and flavonoids in leaves of artichoke and cardoon genotypes grown in floating system. *J. Sci. Food Agric.* 93 1119–1127. 10.1002/jsfa.5861 22936423

[B28] Colla G. Rouphael Y. Jawad R. Kumar P. Rea E. Cardarelli M. (2013b). The effectiveness of grafting to improve NaCl and CaCl2 tolerance in cucumber. *Sci. Hortic. (Amsterdam).* 164 380–391. 10.1016/j.scienta.2013.09.023

[B29] Corrado G. Chiaiese P. Lucini L. Miras-Moreno B. Colla G. Rouphael Y. (2020a). Successive harvests affect yield, quality and metabolic profile of sweet basil (Ocimum basilicum L.). *Agronomy* 10:830. 10.3390/agronomy10060830

[B30] Corrado G. Formisano L. De Micco V. Pannico A. Giordano M. El-Nakhel C. (2020b). Understanding the morpho-anatomical, physiological, and functional response of sweet basil to isosmotic nitrate to chloride ratios. *Biology (Basel).* 9 1–19. 10.3390/biology9070158 32650606 PMC7407521

[B31] Crozier A. Clifford M. N. Ashihara H. (2007). *Plant Secondary Metabolites: Occurrence, Structure and Role in the Human Diet.* Hoboken, NJ: : Wiley-Blackwell, 10.1002/9780470988558

[B32] Cruz L. R. O. Polyzos N. Fernandes Â Petropoulos S. A. Gioia F. Di Dias M. I (2020). Effect of saline conditions on chemical profile and the bioactive properties of three red-colored basil cultivars. *Agronomy* 10:1824. 10.3390/agronomy10111824 PMC7690662 33114065

[B33] das Neves J. P. C. Ferreira L. F. P. Vaz M. M. Gazarini L. C. (2008). Gas exchange in the salt marsh species *Atriplex portulacoides L*. and *Limoniastrum monopetalum L*. in Southern Portugal. *Acta Physiol. Plant.* 30 91–97.

[B34] Dashti A. Khan A. A. Collins J. C. (2009). Effects of salinity on growth, ionic relations and solute content of *sorghum bicolor (L.)* monench. *J. Plant Nutr.* 32 1219–1236. 10.1080/01904160902945333

[B35] David B. Wolfender J. L. Dias D. A. (2015). The pharmaceutical industry and natural products: historical status and new trends. *Phytochem. Rev.* 14 299–315. 10.1007/s11101-014-9367-z

[B36] Fallovo C. Rouphael Y. Cardarelli M. Rea E. Battistelli A. Colla G. (2009a). Yield and quality of leafy lettuce in response to nutrient solution composition and growing season. *J. Food Agric. Environ.* 7 456–462.

[B37] Fallovo C. Rouphael Y. Rea E. Battistelli A. Colla G. (2009b). Nutrient solution concentration and growing season affect yield and quality of *Lactuca sativa* L. var. acephala in floating raft culture. *J. Sci. Food Agric.* 89 1682–1689. 10.1002/jsfa.3641

[B38] Farsaraei S. Moghaddam M. Pirbalouti A. G. (2020). Changes in growth and essential oil composition of sweet basil in response of salinity stress and superabsorbents application. *Sci. Hortic. (Amsterdam).* 271:109465. 10.1016/j.scienta.2020.109465

[B39] Formisano L. Ciriello M. El-Nakhel C. De Pascale S. Rouphael Y. (2021a). Dataset on the effects of anti-insect nets of different porosity on mineral and organic acids profile of *Cucurbita pepo L*. *Fruits Leaves. Data* 6:50. 10.3390/data6050050

[B40] Formisano L. Ciriello M. El-Nakhel C. Kyriacou M. C. Rouphael Y. (2021b). Successive harvests modulate the productive and physiological behavior of three genovese pesto basil cultivars. *Agronomy* 11:560. 10.3390/agronomy11030560

[B41] Gharibzahedi S. M. T. Jafari S. M. (2017). The importance of minerals in human nutrition: bioavailability, food fortification, processing effects and nanoencapsulation. *Trends Food Sci. Technol.* 62 119–132. 10.1016/j.tifs.2017.02.017

[B42] Hegazy M. H. Sabra A. S. Alharbi B. M. Said-Al Ahl H. A. H. Astatkie T. Grulova D. (2019). Ameliorative effects of supplemental nutrition on growth and essential oil yield of saline irrigated satureja montana. *J. Essent. Oil Bearing Plants* 22 1218–1227. 10.1080/0972060X.2019.1682681

[B43] Kampfenkel K. Van Montagu M. Inzé D. (1995). Extraction and determination of ascorbate and dehydroascorbate from plant tissue. *Anal. Biochem.* 225 167–169. 10.1006/abio.1995.1127 7778771

[B44] Karthika K. S. Rashmi I. Parvathi M. S. (2018). “Biological functions, uptake and transport of essential nutrients in relation to plant growth,” in *Plant Nutrients and Abiotic Stress Tolerance*, eds Hasanuzzaman M. Fujita M. Oku H. Nahar K. Hawrylak-Nowak B. (Singapore: Springer Singapore), 1–49. 10.1007/978-981-10-9044-8_1

[B45] Kyriacou M. C. Rouphael Y. (2018). Towards a new definition of quality for fresh fruits and vegetables. *Sci. Hortic. (Amsterdam).* 234 463–469. 10.1016/j.scienta.2017.09.046

[B46] León K. Mery D. Pedreschi F. León J. (2006). Color measurement in L*a*b* units from RGB digital images. *Food Res. Int.* 39 1084–1091. 10.1016/j.foodres.2006.03.006

[B47] Liu Z. X. Bie Z. L. Huang Y. Zhen A. Lei B. Zhang H. Y. (2012). Grafting onto Cucurbita moschata rootstock alleviates salt stress in cucumber plants by delaying photoinhibition. *Photosynthetica* 50 152–160.

[B48] Ma Y. Wang P. Zhou T. Chen Z. Gu Z. Yang R. (2019). Role of Ca2+ in phenolic compound metabolism of *barley (Hordeum vulgare L.)* sprouts under NaCl stress. *J. Sci. Food Agric.* 99 5176–5186. 10.1002/jsfa.9764 31021402

[B49] Marotti M. Piccaglia R. Giovanelli E. (1996). Differences in essential oil composition of basil (*Ocimum basilicum L.*) italian cultivars related to morphological characteristics. *J. Agric. Food Chem.* 44 3926–3929. 10.1021/jf9601067

[B50] Munns R. Tester M. (2008). Mechanisms of salinity tolerance. *Annu. Rev. Plant Biol.* 59 651–681. 10.1146/annurev.arplant.59.032607.092911 18444910

[B51] Nedjimi B. Daoud Y. (2009). Ameliorative effect of CaCl2 on growth, membrane permeability and nutrient uptake in Atriplex halimus subsp. schweinfurthii grown at high (NaCl) salinity. *Desalination* 249 163–166. 10.1016/j.desal.2009.01.019

[B52] Nemat Alla M. M. Abogadallah G. M. Badran E. G. Nada R. M. Hassan N. M. (2014). Supplementary CaCl2 ameliorates wheat tolerance to NaCl. *Acta Physiol. Plant* 36:2103.

[B53] Nicoletto C. Santagata S. Bona S. Sambo P. (2013). Influence of cut number on qualitative traits in different cultivars of sweet basil. *Ind. Crops Prod.* 44 465–472. 10.1016/j.indcrop.2012.10.009

[B54] Niinemets Ü (2010). Mild versus severe stress and BVOCs: thresholds, priming and consequences. *Trends Plant Sci.* 15 145–153. 10.1016/j.tplants.2009.11.008 20006534

[B55] Ntatsi G. Aliferis K. A. Rouphael Y. Napolitano F. Makris K. Kalala G. (2017). Salinity source alters mineral composition and metabolism of Cichorium spinosum. *Environ. Exp. Bot.* 141 113–123. 10.1016/j.envexpbot.2017.07.002

[B56] Parida A. K. Das A. B. (2005). Salt tolerance and salinity effects on plants: a review. *Ecotoxicol. Environ. Saf.* 60 324–349. 10.1016/j.ecoenv.2004.06.010 15590011

[B57] Pathare P. B. Opara U. L. Al-Said F. A. J. (2013). Colour measurement and analysis in fresh and processed foods: a review. *Food Bioprocess Technol.* 6 36–60. 10.1007/s11947-012-0867-9

[B58] Pellegrini N. Re R. Yang M. Rice-Evans C. (1998). Screening of dietary carotenoids and carotenoid-rich fruit extracts for antioxidant activities applying 2,2’-azinobis(3-ethylenebenzothiazoline-6- sulfonic acid radical cation decolorization assay. *Methods Enzymol.* 299 379–389. 10.1016/S0076-6879(99)99037-7

[B59] Petropoulos S. A. Levizou E. Ntatsi G. Fernandes Â Petrotos K. Akoumianakis K. (2017). Salinity effect on nutritional value, chemical composition and bioactive compounds content of *Cichorium spinosum* L. *Food Chem.* 214 129–136. 10.1016/j.foodchem.2016.07.080 27507457

[B60] Poiroux-Gonord F. Bidel L. P. R. Fanciullino A. L. Gautier H. Lauri-Lopez F. Urban L. (2010). Health benefits of vitamins and secondary metabolites of fruits and vegetables and prospects to increase their concentrations by agronomic approaches. *J. Agric. Food Chem.* 58 12065–12082. 10.1021/jf1037745 21067179

[B61] Rengel Z. (1992). The role of calcium in salt toxicity. *Plant. Cell Environ.* 15 625–632. 10.1111/j.1365-3040.1992.tb01004.x

[B62] Rouphael Y. Kyriacou M. C. (2018). Enhancing quality of fresh vegetables through salinity eustress and biofortification applications facilitated by soilless cultivation. *Front. Plant Sci.* 9:1254. 10.3389/fpls.2018.01254 30186305 PMC6113394

[B63] Rouphael Y. Petropoulos S. A. Cardarelli M. Colla G. (2018). Salinity as eustressor for enhancing quality of vegetables. *Sci. Hortic.* 234 361–369. 10.1016/j.scienta.2018.02.048

[B64] Scagel C. F. Bryla D. R. Lee J. (2017). Salt exclusion and mycorrhizal symbiosis increase tolerance to NaCl and CaCl2 salinity in ‘Siam Queen’. *Basil. Hortscience* 52 278–287. 10.21273/HORTSCI11256-16

[B65] Scagel C. F. Lee J. Mitchell J. N. (2019). Salinity from NaCl changes the nutrient and polyphenolic composition of basil leaves. *Ind. Crops Prod.* 127 119–128. 10.1016/j.indcrop.2018.10.048

[B66] Shaw T. M. Moore J. A. Marshall J. D. (1998). Root chemistry of Douglas-fir seedlings grown under different nitrogen and potassium regimes. *Can. J. For. Res.* 28 1566–1573. 10.1139/x98-136

[B67] Singleton V. L. Orthofer R. Lamuela-Raventós R. M. (1999). Analysis of total phenols and other oxidation substrates and antioxidants by means of folin-ciocalteu reagent. *Methods Enzymol.* 299 152–178. 10.1016/S0076-6879(99)99017-1

[B68] Tarchoune I. Baâtour O. Harrathi J. Cioni P. L. Lachaâl M. Flamini G. (2013). Essential oil and volatile emissions of basil (Ocimum basilicum) leaves exposed to NaCl or Na2SO4 salinity. *J. Plant Nutr. Soil Sci.* 176 748–755. 10.1002/jpln.201200278

[B69] Trajkova F. Papadantonakis N. Savvas D. (2006). Comparative effects of NaCl and CaCl2 salinity on cucumber grown in a closed hydroponic system. *HortScience* 41 437–441. 10.21273/hortsci.41.2.437

[B70] Wang X. L. Guo X. L. Hou X. G. Zhao W. Xu G. W. Li Z. Q. (2014). Effects of leaf zeatin and zeatin riboside induced by different clipping heights on the regrowth capacity of ryegrass. *Ecol. Res.* 29 167–180. 10.1007/s11284-013-1107-0

[B71] Was̈kiewicz A. Muzolf-Panek M. Goliński P. (2013). “Phenolic content changes in plants under salt stress,” in *Ecophysiology and Responses of Plants Under Salt Stress*, eds Ahmad P. Azooz M. Prasad M. N. V. (New York, NY: Springer), 283–314. 10.1007/978-1-4614-4747-4_11

[B72] Zahedi S. M. Nabipour M. Azizi M. Gheisary H. Jalali M. Amini Z. (2011). Effect of kinds of salt and its different levels on seed germination and growth of basil plant. *World Appl. Sci. J.* 15 1039–1045.

[B73] Zheljazkov V. D. Cantrell C. L. Tekwani B. Khan S. I. (2008). Content, composition, and bioactivity of the essential oils of three basil genotypes as a function of harvesting. *J. Agric. Food Chem.* 56 380–385. 10.1021/jf0725629 18095647

[B74] Zhou D. Xiao M. (2010). Specific ion effects on the seed germination of sunflower. *J. Plant Nutr.* 33 255–266. 10.1080/01904160903434295

[B75] Zuccarini P. Okurowska P. (2008). Effects of mycorrhizal colonization and fertilization on growth and photosynthesis of sweet basil under salt stress. *J. Plant Nutr.* 31 497–513. 10.1080/01904160801895027

