# Supplementary material for: Differential Response to NaCl Osmotic Stress in Sequentially Harvested Hydroponic Red and Green Basil and the Role of Calcium
Source: Front Plant Sci. 2022 Mar 9;13:799213. doi: 10.3389/fpls.2022.799213 (PMC8959763; doi:10.3389/fpls.2022.799213)
Supplement: Supplementary file 1 [file Table_1.docx]

Supplementary Material

**Table S1.** List of identified volatile compounds (VOCs). Compounds were sorted according to retention time.

| **Compound** | **Retention time (min)** | **Compound Class** | **PubChem CID** |
| --- | --- | --- | --- |
| acetic acid methyl ester | 10.561 | Aliphatic esters | 6584 |
| Ethyl acetate | 10.526 | Aliphatic esters | 8857 |
| methyl alcohol | 10.862 | Aliphatic alcohols | 887 |
| 2-Methylbutyraldehyde | 11.555 | Aliphatic aldehydes | 7284 |
| 3-Methylbutanal | 11.714 | Aliphatic aldehydes | 11552 |
| Ethanol | 12.160 | Aliphatic alcohols | 702 |
| Ethyl propionate | 13.253 | Aliphatic esters | 7749 |
| n-Propyl acetate | 13.993 | Aliphatic esters | 7997 |
| Methyl butyrate | 14.489 | Aliphatic esters | 12180 |
| α-Pinene | 15.994 | Monoterpene hydrocabons | 6654 |
| α-Thujene | 16.171 | Monoterpene hydrocabons | 17868 |
| Ethyl butyrate | 16.661 | Aliphatic esters | 7762 |
| Toluene | 17.064 | Aromatic compounds | 1140 |
| Camphene | 18.072 | Monoterpene hydrocabons | 6616 |
| Hexanal | 18.908 | Aliphatic aldehydes | 6184 |
| β-Pinene | 20.062 | Monoterpene hydrocabons | 14896 |
| β-Phellandrene | 20.639 | Monoterpene hydrocabons | 11142 |
| Ethylbenzene | 21.106 | Aromatic componds | 7500 |
| o-Xylene | 21.802 | Aromatic componds | 7237 |
| 1-Penten-3-ol | 21.973 | Aliphatic alcohols | 12020 |
| 2-Methoxyfuran | 22.237 | Aromatic componds | 117476 |
| β-Myrcene | 22.424 | Monoterpene hydrocabons | 31253 |
| α-Phellandrene | 22.735 | Monoterpene hydrocabons | 7460 |
| α-Terpinene | 23.406 | Monoterpene hydrocarbons | 7462 |
| p-Xylene | 23.865 | Aromatic componds | 7809 |
| D-Limonene | 24.307 | Monoterpene hydrocarbons | 440917 |
| 2-Hexenal | 24.515 | Aliphatic aldehydes | 5281168 |
| Eucalyptol | 24.837 | Oxygenated monoterpene | 2758 |
| 2-Hexenal | 25.315 | Aliphatic aldehydes | 5281168 |
| (E)-β-Ocimene | 25.616 | Monoterpene hydrocarbons | 5281553 |
| (Z)-β-Ocimene | 26.418 | Monoterpene hydrocarbons | 5320250 |
| 3-Octanone | 26.691 | Aliphatic ketones | 246728 |
| Hexyl acetate | 26.780 | Aliphatic esters | 8908 |
| Styrene | 27.040 | Aromatic componds | 7501 |
| M-Cymene | 27.565 | Monoterpene hydrocarbons | 10812 |
| cis-4-Carene | 28.049 | Monoterpene hydrocarbons | 21674939 |
| Octanal | 28.210 | Alyphatic aldehydes | 454 |
| Cyclopropanecarboxaldehyde, 2-methyl-2-(4-methyl-3-pentenyl) | 28.734 | Alyphatic aldehydes | 59018633 |
| cis-2-Penten-1-ol | 29.043 | Aliphatic alcohols | 5364919 |
| cis-3-Hexenyl acetate | 29.211 | Aliphatic esters | 5363388 |
| 1-Hexanol | 30.330 | Aliphatic alcohols | 8103 |
| trans-3-Hexenol | 30.859 | Aliphatic alcohols | 5284503 |
| Octen-1-ol acetate | 31.559 | Aliphatic esters | 520583 |
| Allo-Ocimene | 31.662 | Monoterpene hydrocarbons | 5368821 |
| cis-3-Hexen-1-ol | 31.782 | Aliphatic alcohols | 5281167 |
| 3-Octanol | 31.924 | Aliphatic alcohols | 11527 |
| trans-2-Hexen-1-ol | 32.608 | Aliphatic alcohols | 5318042 |
| 2,4-Hexadienal | 33.282 | Alyphatic aldehydes | 637564 |
| 1-Octen-3-ol | 34.226 | Aliphatic alcohols | 18827 |
| p-Mentha-1,3,8-triene | 34.840 | Monoterpene hydrocarbons | 176983 |
| α-cubebene | 35.176 | Sesquiterpene hydrocarbons | 86609 |
| Octyl acetate | 35.480 | Aliphatic esters | 8164 |
| cis-Linalool Oxide | 35.581 | Oxygenated monoterpenes | 91752909 |
| Fenchyl acetate | 35.750 | Aliphatic esters | 107217 |
| α-Copaene | 36.763 | sesquiterpenes hydrocarbons | 442355 |
| α-Cedrene | 37.519 | sesquiterpenes hydrocarbons | 442348 |
| 3,5-Octadien-2-one | 37.634 | Aliphatic ketones | 5352876 |
| β-Linalool | 38.037 | Oxygenated monoterpenes | 6549 |
| Camphor | 38.215 | Oxygenated monoterpenes | 2537 |
| 1-Octanol | 38.334 | Aliphatic alcohols | 957 |
| α-Bergamotene | 39.398 | sesquiterpene hydrocarbon | 86608 |
| α-Santalene | 39.646 | sesquiterpene hydrocarbon | 94164 |
| Fenchyl alcohol | 39.840 | Oxygenated monoterpenes | 6997371 |
| trans-α-Bergamotene | 40.030 | sesquiterpene hydrocarbons | 6429302 |
| α-Guaiaene | 40.404 | sesquiterpene hydrocarbons | 5317844 |
| Terpinen-4-ol | 40.660 | Oxygenated monoterpenes | 11230 |
| Caryophyllene | 40.975 | sesquiterpene hydrocarbons | 5281515 |
| β-Patchoulene | 41.322 | sesquiterpene hydrocarbons | 101731 |
| 1-epi-Bicyclosesquiphellandrene | 42.261 | Monoterpene hydrocarbons | 521496 |
| β-Farnesene | 42.393 | sesquiterpene hydrocarbons | 5281517 |
| α-Terpineol | 42.559 | Aromatic compounds | 17100 |
| α-Caryophyllene | 43.563 | sesquiterpene hydrocarbons | 23204 |
| p-Menth-1-en-3,8-diol, trans | 43.823 | Oxygenated monoterpenes | 6431168 |
| (-)-Borneol | 44.187 | Oxygenated monoterpene | 1201518 |
| Aciphyllene | 44.419 | sesquiterpene hydrocarbons | 565709 |
| α-Bulnesene | 44.889 | sesquiterpene hydrocarbons | 6432384 |
| Eudesma-4(14),11-diene | 45.330 | sesquiterpene hydrocarbons | 6432497 |
| Cadina-1(10),4-diene | 46.180 | sesquiterpene hydrocarbons | 10223 |
| γ-Muurolene | 46.450 | sesquiterpene hydrocarbons | 12313020 |
| Nerol | 46.954 | Oxygenated monoterpenes | 643820 |
| α-Muurolene | 47.485 | sesquiterpene hydrocarbons | 12306047 |
| trans-Geraniol | 48.338 | Oxygenated monoterpenes | 637566 |
| trans-Calamenene | 48.874 | sesquiterpene hydrocarbons | 6429022 |
| Eugenol | 58.561 | Aromatic compounds | 3314 |
